# Effects of Isoflurane Anesthesia on Ensemble Patterns of Ca^2+^ Activity in Mouse V1: Reduced Direction Selectivity Independent of Increased Correlations in Cellular Activity

**DOI:** 10.1371/journal.pone.0118277

**Published:** 2015-02-23

**Authors:** Pieter M. Goltstein, Jorrit S. Montijn, Cyriel M. A. Pennartz

**Affiliations:** 1 Center for Neuroscience, Swammerdam Institute for Life Sciences, University of Amsterdam, Amsterdam, The Netherlands; 2 Research Priority Program Brain and Cognition, University of Amsterdam, Amsterdam, The Netherlands; Monash University, AUSTRALIA

## Abstract

Anesthesia affects brain activity at the molecular, neuronal and network level, but it is not well-understood how tuning properties of sensory neurons and network connectivity change under its influence. Using in vivo two-photon calcium imaging we matched neuron identity across episodes of wakefulness and anesthesia in the same mouse and recorded spontaneous and visually evoked activity patterns of neuronal ensembles in these two states. Correlations in spontaneous patterns of calcium activity between pairs of neurons were increased under anesthesia. While orientation selectivity remained unaffected by anesthesia, this treatment reduced direction selectivity, which was attributable to an increased response to the null-direction. As compared to anesthesia, populations of V1 neurons coded more mutual information on opposite stimulus directions during wakefulness, whereas information on stimulus orientation differences was lower. Increases in correlations of calcium activity during visual stimulation were correlated with poorer population coding, which raised the hypothesis that the anesthesia-induced increase in correlations may be causal to degrading directional coding. Visual stimulation under anesthesia, however, decorrelated ongoing activity patterns to a level comparable to wakefulness. Because visual stimulation thus appears to ‘break’ the strength of pairwise correlations normally found in spontaneous activity under anesthesia, the changes in correlational structure cannot explain the awake-anesthesia difference in direction coding. The population-wide decrease in coding for stimulus direction thus occurs independently of anesthesia-induced increments in correlations of spontaneous activity.

## Introduction

Anesthesia can be used to study changes in information processing accompanying the loss of consciousness [1,2]. General anesthesia impairs activity in, and functional long-range connectivity between, frontal, parietal and posterior regions of the human brain [3,4] and may reduce the capacity for information-integration [5,6]. Also the local dynamics of cortical spontaneous activity have been shown to differ between the awake and anesthetized state [7–10], but it remains unclear how these changes affect single-neuron and population coding, for instance of orientation and direction of movement in the primary visual cortex.

General anesthesia induces pronounced changes in receptor, ion channel, single neuron, network and behavioral function [11,12]. At the molecular level, a common denominator in the function of most anesthetics is a potentiation of GABA_A_ receptor activity and an inhibition of Potassium channels and AMPA- and NMDA receptors [1,12–14]. Neuromodulators and presynaptic effects mediate additional anesthesia-induced changes in global brain states [15,16]. A striking feature of the neocortex under deep levels of anesthesia is the emergence of a slow rhythm of alternating depolarized (UP) and hyperpolarized (DOWN) states that entrains spontaneous action potential firing and results in strongly synchronized activity patterns [7,17]. Given that two-photon calcium imaging has been validated using single cell electrophysiology, this technique has revealed that also under light levels of anesthesia, and in the absence of clear-cut UP/DOWN states, correlations between spontaneous activity patterns of individual neurons increase [8,18].

Increased correlated activity can lead to a reduction in population-coded mutual information about stimuli by rendering the cortical code redundant [19–22]. Furthermore, it may diminish the number of discriminative states the neocortex can assume, which has been theorized to accompany loss of consciousness [1,2,23]. Accordingly, sensory stimulation in the awake state has been associated with decorrelation of activity patterns of populations of cortical neurons [24–26]. Correlated activity can, however, also have a different effect on target structures and improve fidelity of stimulus coding as compared to uncorrelated patterns [27–32].

Effects of light anesthesia on receptive field properties in auditory, somatosensory and olfactory structures indeed suggest increased redundancy of single-neuron firing patterns [33–36], while deeper levels of anesthesia may result in reduced amplitude of stimulus evoked responses in somatosensory thalamus and cortex [37,38] and visual cortex [39–41]. Recent studies of Haider et al. [42], Adesnik et al. [43] and Vaiceliunaite et al. [44] in primary visual cortex (V1) suggest that these changes may result from weakened inhibition and surround suppression during anesthesia. Orientation tuning parameters of V1 neurons, however, fell within the same range of values for experiments done in awake and anesthetized animals [21,45–53]. On the other hand, anesthesia does affect more complex receptive field properties and attentional top-down mechanisms in visual cortex (e.g. [54,55]).

To compare the awake and anesthetized state more directly, we used in vivo two-photon calcium imaging of the same neurons with and without isoflurane anesthesia. First, we studied whether orientation and direction coding are changed at the single-cell and population level, and second, we asked whether changes in direction coding can be explained by increased redundancy, as gauged by correlations in activity patterns.

## Materials and Methods

### Animal preparation for imaging

All animals experiments were conducted after approval by the ethical committee (DEC) of the University of Amsterdam (Protocol number: DED162). Using dental cement, nine adult male C57Bl/6JOlaHsd mice (Harlan) were fitted with a cranial bar and allowed one to three weeks accommodation time before imaging. Although Ranson et al. [56] showed that C57Bl/6JOlaHsd mice have specific defects in homeostatic visual cortex plasticity it is unlikely that these defects provide an alternative explanation for the results of our study. On the morning of a calcium-imaging experiment, buprenorphine (0.05 mg/kg bodyweight) was injected subcutaneously half an hour before induction of general anesthesia using isoflurane (3.0% in 100% O_2_). While performing the craniotomy and during dye-loading, anesthesia was maintained at an isoflurane concentration ranging between 1.0% and 1.5% in 100% O_2_. A craniotomy was made above the stereotactic location of V1 while leaving the dura mater intact. Precise coordinates were -4.0 mm from Bregma, 2.5 mm lateral [57], a region of V1 that responds to stimuli presented at approximately 45 degrees Azimuth and 10 degrees elevation [58], which corresponds to the center position of the screen used for visual stimulation. The craniotomy was kept wet by applying pH buffered artificial cerebrospinal fluid (aCSF: 125 mM NaCl, 5 mM KCl, 1.3 mM MgSO_4_ ·7H_2_O, 2.0 mM NaH_2_PO_4_, 2.5 mM CaCl_2_ ·2H_2_O, 10 mM Glucose and 10 mM HEPES in distilled water, pH adjusted to 7.4; [59]). During the entire procedure, the eyes were kept moist with eye-drops and protected from bright light.

### Two-photon calcium imaging

Multi-cell bolus loading of neurons in the primary visual cortex with the fluorescent calcium indicator Oregon Green BAPTA-1 AM (OGB) and Sulforhodamine 101 (SR101) was done as previously described [60–62]. In brief, 1μl OGB stock solution (50μg OGB dissolved in 4μl dimethylsulphoxide with 20% pluronic acid) was mixed with 20μl pipette solution (150 mM NaCl, 2.5 mM KCl and 10 mM HEPES in distilled water, pH adjusted to 7.4) and 4μl SR101 stock solution (500μg SR101 in 500μl pipette solution, diluted with pipette solution by a 50:1 ratio), reaching a final concentration of 0.5 mM OGB and 5 to 10 μM SR101, and injected in V1 at a depth of 200 to 300 μm below the cortical surface by applying 0.7 bar pressure on the back of a glass pipette (4 to 6 MΩ resistance) for 120 to 180 s. After dye-loading, the exposed area of the brain was sealed with agarose (1.5% in aCSF) and a cover glass that was secured using cyanoacrylate glue.

Spontaneous and evoked calcium activity of layer 2/3 neurons was imaged using a Leica SP5 confocal system modified for in vivo two-photon laser scanning microscopy with a Spectra-Physics Mai-Tai HP laser, set to an excitation wavelength of 810 nm. Images of 256 × 256 pixels were acquired at 10 to 17 frames per second using two non-descanned photo-multiplier tubes, which were band-pass filtered for 500 to 550 nm (OGB) and 565 to 605 nm (SR101).

The order of the experimental conditions (‘Awake’ and ‘Anesthesia’) was counterbalanced across recordings to prevent bias originating from a sequence effect. Before the onset of recordings in the awake condition, the mouse was allowed to recover from anesthesia for approximately 30 to 60 minutes, whereas the animal was kept under anesthesia (1.0% isoflurane, 20 to 30 minutes) before the start of anesthetized recordings. To ensure delivery of a stable concentration of isoflurane, we used the calibrated, commercially available, Vapomatic isoflurane vaporizer (A.M. Bickford, Inc., NY, USA). The anesthetic gas mixture flowed at a rate of 500 ml per minute into a small plastic nose cone that fitted closely around the nose and mouth area of the mouse. Given that the respiratory volume for C57Bl6 mice lies around 30 ml/ minute ([63]; see also [64]) and that the residual volume in the nose cone was less that 2 ml, the flow rate of the anesthetic gas (at 500 ml/minute) ensured a constant concentration of isoflurane being delivered to the mouse. Anesthesia was subsequently verified by absence of the hind-paw and eyelid reflex. During the entire experiment, we regularly inspected both eyes for cataracts and checked whether the eyelids were properly open. In the anesthesia condition, we additionally provided eye drops to keep the eyes slightly moist. In both experimental conditions (awake and anesthesia), spontaneous calcium activity was measured first, with the display showing a uniform grey image (isoluminant to the moving gratings), followed by visual stimulation (‘evoked activity’).

Visual stimulation was done by presenting full-field moving square-wave gratings (100% contrast, 0.05 cycles per degree and 2 cycles per second drifting in 8 directions for 8 trials each) using a Dell workstation with a 15” TFT screen running MatLab (The Mathworks) and Psychophysics Toolbox (http://www.psychtoolbox.org), positioned 20 cm from the eye. The center of the screen was (with respect to the eye) at 45 degrees Azimuth and 10 degrees elevation. The area of visual stimulation covered approximately 74 degrees of horizontal visual space and 60 degrees of vertical visual space. Given the size of the screen and the approximately 10–20 degree median diameter of the suprathreshold receptive fields in mouse V1 [65,66], the majority of cells in our experiment are estimated to have had their full receptive field stimulated by the screen (as has been argued similarly in e.g. [67–69]).

### Data preprocessing

Small movement artifacts in the x-y plane were corrected for by realigning the images [70]. Recordings with clear movement artifacts along the z-axis were rejected. Regions of interest (ROIs) were outlined semi-automatically for all cells (neurons and astrocytes), bloodvessels and neuropil (which was defined as the area in the image devoid of cells and bloodvessels). Time series were constructed for all ROIs by averaging the fluorescence within their borders for each frame.

Time series of identified neurons were corrected for possible contamination by non-cell-specific fluorescence signals in the neuropil using standard methods for OGB imaging, previously described by Kerlin et al. [71]. In brief, a local neuropil signal was calculated in a region between 2 to 5 μm around the cell or blood vessel of interest. Next, the ratio of neuropil signal leaking into the ROIs was estimated by calculating the ratio between average blood vessel-fluorescence and average surrounding neuropil-fluorescence. Blood vessels, having the size of neurons, were selected for calculating this contamination ratio. Green fluorescence (500–550 nm) emitted from within blood vessels should on average be approximately zero. Any detectable level of fluorescence in blood vessels can therefore be ascribed to neuropil contamination, allowing calculation of the fraction of neuropil signal ‘leaking’ into the blood vessel. Ideally, the contamination ratio is zero, but in these experiments the values ranged between 0.59 and 0.83, which is slightly higher than in Kerlin et al. [71]. Corrected time series were subsequently achieved by subtracting the surrounding neuropil signal from the raw fluorescence time series of each neuron, scaled by the estimated neuropil contamination ratio.

A number of previous studies have shown that there exists a strong and almost linear correlation between ΔF/F responses in somatic calcium imaging time series (using OGB as calcium indicator) and spiking activity of single neurons (e.g. [8,60,67,72,73]). The neuropil corrected ΔF/F time series were therefore interpreted as time series reflecting spiking activity, low-pass filtered by the time constant of the calcium transient and with symmetric (Gaussian) random noise added [74]. In this study, fluorescence signals were not directly validated against electrophysiological measurements of action potential activity. Analysis of the ΔF/F time series, however, proved to reproduce a number of previously reported electrophysiological findings, which supports the validity of the calcium imaging approach. These findings include anesthesia-induced changes in correlations and coherence of spontaneous cellular activity patterns (see Results section) as measured using electrophysiology [7] and electrophysiology-validated calcium imaging [8,18]. In addition, we observed anesthesia independent orientation selectivity (see Results section) as reported using electrophysiology by Ikeda and Wright [39] and movement-induced potentiation of visual responses (see Results section), as reported in single-unit recordings by Niell & Stryker [51]. The ability to reproduce these earlier findings provides a further validation of the approach.

### Analysis of spontaneous calcium activity

For periods of spontaneous activity, ΔF/F values were calculated for each frame *t* by dividing the absolute fluorescence *F*_*t*_ by the mean of the lowest 50% fluorescence values in a 20 s window before frame *t* [8,18]. The correlation in calcium activity between a pair of neurons was determined by calculating the Pearson correlation coefficient of the two z-scored ΔF/F time series for the entire segment of spontaneous activity (which lasted at least 200 seconds). Spectral analyses were conducted using the Fieldtrip toolbox (http://www.ru.nl/donders/fieldtrip; [75]). Power spectra were calculated for the entire period of spontaneous activity using a sliding window and single Hanning taper. Because of the slow temporal dynamics of the calcium signal (τ ≈ 500 ms), we did not expect to resolve large signal-related differences in frequencies above 3 Hz and therefore normalized the spectra by dividing them over the mean power in the 3.5–4.0 Hz frequency band, which was still well below the Nyquist frequency of 5 Hz or higher. Cross-spectrum coherence of calcium signals was calculated similarly, but using a multi-taper method and spectral smoothing of 0.1 Hz.

### Orientation tuning

Single trial ΔF/F responses were defined as the mean percentage increase in fluorescence during visual stimulation (0.1 to 3.0 s after stimulus onset) from baseline (mean fluorescence in the period of -2 to -0.1 s before stimulus onset) and converted to a vector in orientation and direction space where the angle equals stimulus orientation or direction *θ* and the length equals the magnitude of the single trial response *R*_*θj*_ where *j* indicates trial number. Neurons were classified as significantly orientation- or direction-tuned when the mean of the *R*_*θj*_-vector (across the awake and anesthetized condition) was significantly different from zero, tested with a Hotellings T^2^ test p < 0.05 [76,77]. We opted for circular statistics (Hotelling’s T^2^ test) because this method assumes a circular relation between the movement orientations or directions and therefore increases the chance to exclude cells that respond to, for instance, two orthogonal orientations (for which it would not make sense to quantify orientation tuning parameters). Here, the term “orientation tuning” is used as classification for neurons showing at least orientation-selective, but possibly also direction-selective, responses to moving gratings. Similar results were obtained when the classification was done separately for awake and anesthetized conditions.

By selecting cells that were significantly tuned in both conditions, we rejected a relatively large number of neurons that did not show reliable stimulus-induced responses across both anesthetized and awake recordings (577 out of 687 cells). The magnitude of this number of rejections can be ascribed, first, to the long time span of the experiments, ranging across many hours and given the transient nature of OGB-labeling. In addition to both imaging epochs, this time span included periods of recovery from anesthesia and additional recordings of spontaneous activity, not included in this study. Secondly, we only included neurons that were significantly orientation-tuned in both states. A relatively large group (N = 87) was only visually responsive and orientation-tuned in one state but not the other. These cells were excluded because one needs to obtain orientation-tuned responses from both states in order to quantify and compare tuning curve parameters. When including these cells, however, 29% of the total population was significantly orientation-tuned in at least one (awake/anesthesia) state.

Tuning curves were constructed by fitting the average response to the stimulus directions with a two peaked 360 degree wrapped Gaussian curve (Eq. 1; [76]) for the awake and anesthesia condition separately. *R*_*offset*_ is the baseline fluorescence, *R*_*pref*_ the response to the preferred direction *θ*_*pref*_, *R*_*null*_ the response to the opposite direction (i.e. null direction; *θ*_*pref*_ + 180°) and σ the standard deviation of the Gaussian distribution function. The function *y = ang(x)* wraps the angular difference *x* = *θ* − *θ*_*pref*_ on an interval between 0° and 180°.

$$R_{\vartheta }=R_{offset}+R_{pref}\cdot e^{-\frac{ang{\left(\vartheta -\vartheta_{pref}\right)}^{2}}{2\sigma^{2}}}+R_{null}\cdot e^{-\frac{ang{\left(\vartheta +180-\vartheta_{pref}\right)}^{2}}{2\sigma^{2}}}$$ Eq 1

The cellular response to each movement direction was measured from the fitted tuning curve with a 1° angular resolution. Tuning curve bandwidth was determined as half width at 1/√2 maximum of the tuning curve [78]. The orientation selectivity index (OSI) was calculated by dividing the difference between the response to the preferred orientation (defined as the mean of the preferred direction- and the null direction response, R_pref,ori_) and the mean of the orthogonal orientations, by the sum of these, (*R*_*pref,ori*_ -*R*_*ortho,ori*_)/(*R*_*pref,ori*_ +*R*_*ortho,ori*_). Similarly, the direction selectivity index (DSI) was calculated by dividing the difference between the response to the preferred direction and the null direction by the sum of these responses, (*R*_*pref,dir*_—*R*_*null,dir*_)/(*R*_*pref,dir*_ + *R*_*null,dir*_) (e.g. [51,68,79]).

The effectiveness of tuning curve fitting was addressed using simulation of single trial responses of single cells, derived from 500 ideal tuning curve functions with variable parameters and added noise. Signal strength was defined as response amplitude to the preferred direction. Added noise followed a zero mean Gaussian distribution with a standard deviation of 2, 1, 0.5, 0.25 or 0.125 times the signal amplitude, resulting in a signal to noise ratio of 0.5, 1, 2, 4 and 8. For each ideal tuning curve, 10 raw tuning curve measurements each were simulated by sampling responses to eight directions eight times and averaging these simulated responses of the raw tuning curve. Each raw curve was subsequently fitted using a two-peaked Gaussian function (see above). The properties preferred direction, preferred orientation, OSI, bandwidth and DSI were quantified for both raw- and fitted curves. The variance of the tuning curve parameters of the 10 raw and fitted measurements per ideal (true) tuning curve was used to compute the measurement estimation error. 95% Confidence intervals were calculated using bootstrap resampling (1000 resampling iterations).

### Sparseness

A sparseness measure from Willmore and Tolhurst [80], which was based on the Treves and Rolls [81] measure and made appropriate for responses ranging from −∞ to ∞ (Eq. 2), was adopted to quantify stimulus selectivity of the responses per neuron without assuming a relation between stimuli (e.g. orientation or direction of movement).

$$S_{sn_{i}}=1-\frac{{\left(\sum_{j=1}^{N}\left|r_{ij}\right|/N\right)}^{2}}{\sum_{j=1}^{N}\left(r_{ij}{}^{2}/N\right)}$$ Eq 2

For single-neuron response sparseness of the tuning curves *S*_*sn i*_, *N* represents the number of stimulus directions and *r*_*ij*_ the mean response of a single neuron *i* to a stimulus *j*. Single neuron response sparseness of *1*-(1/*N*) indicates 100% selectivity for a single directional stimulus, while 0 suggests no stimulus selectivity at all.

Population sparseness, *S*_*pop i*_, indicates how sparsely stimuli are coded across groups of neurons. *S*_*pop i*_ is also calculated using equation 2, but now *N* represents the number of neurons and r_*ij*_ the mean response of a single neuron *j* to stimulus *i*. The population sparseness measure approximates *1*-(1/*N*) when only a selective subset of neurons responds to a stimulus and approaches 0 when most neurons respond in a similar fashion to stimuli. Population sparseness was calculated across all recorded neurons in a session for each stimulus direction separately and averaged over stimulus directions.

### Mutual information

Stimulus direction was decoded from single trial (Δ*F*/*F*)_*i*_ responses using a Euclidean distance metric (e.g. [82]) per pair of stimuli. In brief, responses to two stimuli in one of the eight trial blocks were selected for decoding and removed from the dataset. Subsequently, we calculated the Euclidean distance between population response vectors of all of the remaining (seven) trials and the population response vector of the currently decoded trial. For each of the trials, the decoder returned the stimulus that, on average, had the most similar population response vectors compared to the population response vector of the decoded trial (smallest Euclidean distance).

Decoder output was used to calculate how much information calcium responses convey about stimulus orientation and direction. Using the Panzeri-Treves toolbox [83], mutual information was calculated for each of the randomly sampled populations using direct entropy estimation (direct method) and corrected for bias with the ‘Panzeri-Treves’ bias correction algorithm. Because of the number of trials per stimulus (N = 8), a remaining bias could still result in an overestimation of the entropy. Therefore, we performed an additional correction using a bootstrap subtraction method, where we subtracted the average mutual information estimate after a stimulus-randomization procedure (500 samples) from the data-obtained estimate of mutual information. Because the entropy-overestimation is stronger for higher entropies, and entropy in a stimulus-randomized response distribution can be considered maximal compared to a non-randomized distribution, we expect our results to underestimate rather than overestimate mutual information after applying bootstrap subtraction. Nevertheless, any effect of bias should be similar in strength for all experimental conditions and will therefore not affect the comparisons within this study. Using the above described method, mutual information was sampled for 2000 randomly selected groups of 1 to 64 neurons and averaged for each unique stimulus combination.

### Linear regression model

A linear model was implemented to assess the impact of changes in tuning curve parameters and correlations in activity patterns on the amount of mutual information conveyed by those activity patterns. Mutual information was calculated as described above for all possible simultaneously recorded triplets of neurons and for both the awake and anesthetized state. The ensemble size (N = 3) was chosen to allow large enough variation between sampled populations in predictor parameters while having enough neurons to detect changes in redundancy of response patterns and having few enough neurons to avoid regression towards the mean. This approach resulted in a dataset containing 5275 unique triplets of simultaneously recorded neurons from 9 recording sessions. Variables predictive of mutual information were: Mean tuning curve bandwidth, mean orientation selectivity index, mean direction selectivity index, mean ΔF/F response to the preferred direction, mean ΔF/F response to the null direction, mean pairwise correlation between ΔF/F time series recorded during spontaneous activity and mean pairwise correlations recorded during visual stimulation (where the mean refers to the average across the three neurons of the triplet). Variables were represented as differences between the awake and anesthetized state. The dependent variable for regression analysis was the difference in mutual information between the awake and anesthetized state for each of the triplets of neurons. Regression was done by fitting a model predicting linear interactions between each of the predictor variables and a constant term to the dependent variable. Prediction strengths of parameters were expressed in beta-weights. P-values were corrected for multiple comparisons. Whiskers in the bar-plots represent 99% confidence intervals.

### Statistics

All data are represented as mean ± standard error of the mean (SEM) unless stated otherwise. Differences in correlations, tuning properties and response sparseness were tested using the Wilcoxon’s matched pairs signed rank (WMPSR) test. Covariation between these variables was tested using the Pearson correlation coefficient. Decoding performance and mutual information were averaged (± SEM) across unique stimulus combinations with the same angular difference between stimulus directions and tested using a two-way and three-way Anova.

## Results

### Two-photon calcium imaging of visual cortex neurons in the awake and anesthetized state

Using two photon laser scanning microscopy we imaged spontaneous and visually evoked calcium activity in V1 neurons of 9 C57Bl/6JOlaHsd mice during wakefulness and under anesthesia. Neurons and astrocytes were loaded with the fluorescent calcium indicator Oregon Green BAPTA1-AM and the astrocyte marker Sulforhodamine 101 during a brief surgery preceding the experiments [60–62,84]. Previous studies have demonstrated the tight relation between somatic calcium transients and electrophysiologically recorded spikes (e.g. [8,67,71–73]). The experimental conditions ‘Awake’ and ‘Anesthesia’ were counterbalanced across recording sessions to prevent bias originating from a sequence effect (5 awake-first sessions; 5 anesthesia-first sessions; in one mouse, two sequences were run). In both conditions (awake and anesthesia), spontaneous activity was measured first, with a dimly lit grey screen placed in the monocular visual field of the mouse. Visual stimulation was subsequently delivered by presenting full-field moving gratings in 8 directions, each for about 3 s and repeated 8 times on the same screen.

**Fig 1:**
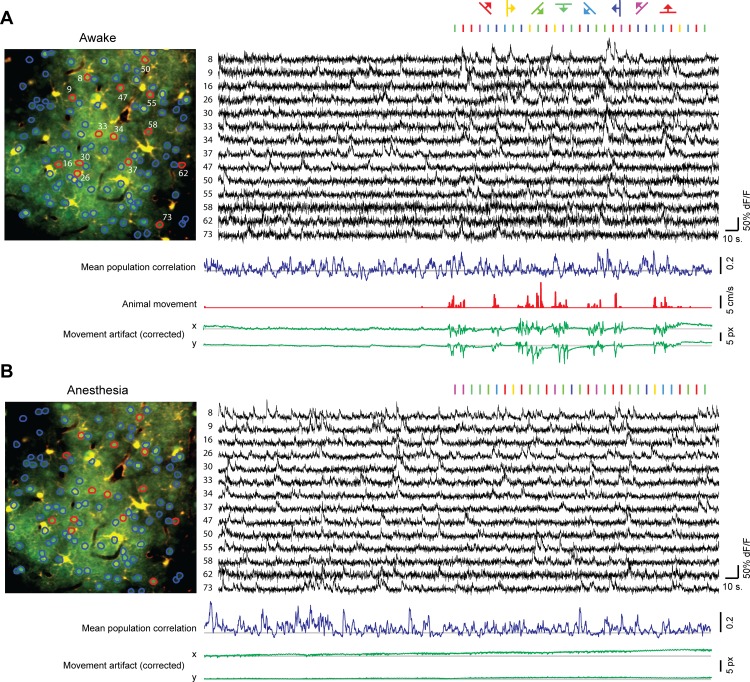
Imaging spontaneous and evoked calcium activity of the same identified neurons during the awake and anesthetized state. (A) Image of detected (red and blue encircled) neurons and astrocytes in the awake mouse. Black traces (right) are the neuropil contamination-corrected ΔF/F calcium time series of the red-encircled neurons in the image (left). Vertical lines above the calcium time series indicate times when visual stimuli (gratings) were presented, each movement direction in a different color. The blue trace represents the evolution of the mean correlation between all pairs of displayed neurons (calculated over a sliding two-second window). The red trace shows animal movement, measured from treadmill motion. The green traces indicate the (corrected) horizontal (x) and vertical (y) image displacement, which was highly correlated with animal motion. Respective scale bars are located on the right, grey horizontal lines represent the horizontal zero axis. (B) Idem as in A, but recorded 45 to 70 minutes later under anesthesia (1.0% isoflurane in O2). Note the difference in scaling of the ΔF/F signals with (A).

In ten recordings, 687 neurons (approximately two thirds of the total number of recorded neurons) could be identified both in the awake condition as well as in the subsequent or preceding anesthesia condition (Fig. 1; on average 68.7 ± 26.2 (SD) neurons per mouse, with a minimum of 36 and a maximum of 111). Periods of spontaneous activity were on average 248 ± 52 s long (min–max: 198–327 s), periods during which activity was evoked visually 393 ± 40 s (min–max: 299–417 s), with a mean sampling frequency of 10.9 ± 2.3 Hz (min–max: 9.8–17.1 Hz) and a mean recording depth of 229μm ± 69μm (min–max: 160–400 μm), corresponding to cortical layer II/III in the mouse. Despite some variation in the above-mentioned parameters between animals, analyzed recordings of each individual mouse had identical length and sampling frequencies in the awake and anesthetized recording. Neuronal calcium signals were extracted from manually defined ROIs and the resulting single-neuron fluorescence time series were corrected for contamination by neuropil fluorescence using a subtraction method described by Kerlin et al. [71].

### Correlations between spontaneous patterns of calcium signals

**Fig 2:**
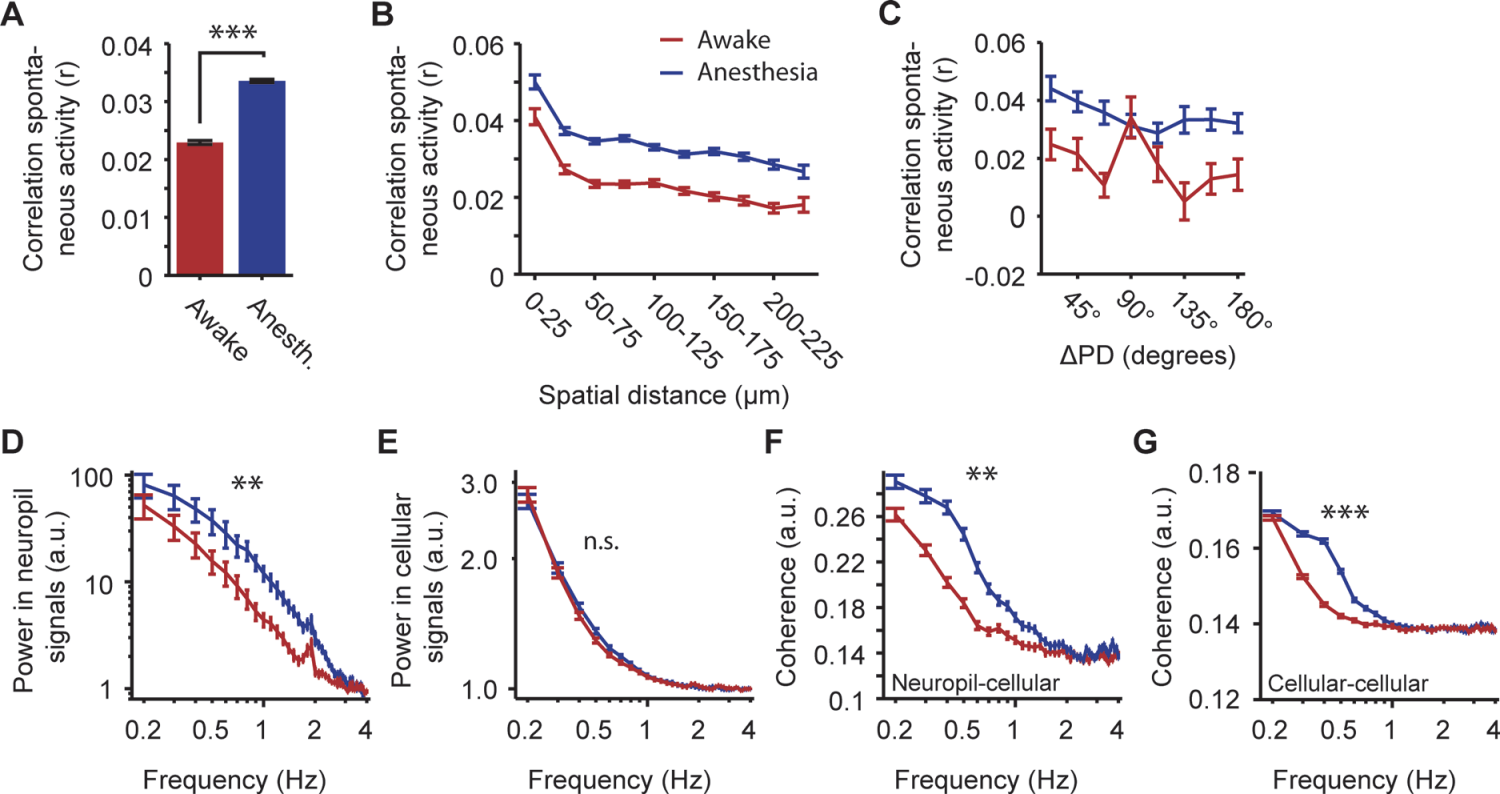
Correlations in spontaneous patterns of calcium activity. (A) Bars show mean pairwise correlation between calcium time series of all simultaneously recorded pairs of neurons. (B) Pairwise correlations as a function of spatial distance between cell bodies (Red: Awake, r = -0.061, p < 10^–10^; Blue: Anesthesia, r = -0.073, p < 10^–10^; n = 26350). (C) Pairwise correlations in spontaneous activity as a function of similarity in preferred direction of orientation-tuned cells (Red: Awake, r = -0.07, p = 0.04; Blue: Anesthesia, r = -0.09, p = 0.008; n = 846). (D) Power spectrum of oscillations in the baselined ΔF/F neuropil signal. (E) Power spectrum of oscillations in neuropil contamination-corrected ΔF/F calcium time series of all simultaneously recorded neurons. (F) Cross-spectrum coherence between neuropil contamination corrected ΔF/F calcium time series of all neurons and the simultaneously recorded, baselined ΔF/F neuropil signal. (G) Cross-spectrum coherence between neuropil contamination corrected ΔF/F calcium time series of all simultaneously recorded pairs of neurons. For each plot, the red line shows data recorded in awake mice, the blue line data from recordings under anesthesia. All values are mean ± SEM, ** p < 0.001, *** p < 10^–7^.

We first verified whether correlations between spontaneous activity patterns of neurons and neuropil were sensitive to the awake-anesthesia manipulation. Pairwise correlations in spontaneous neuronal spiking activity have been found to be stronger under anesthesia compared to the awake state [8,18]. In this study, a similar effect was observed for correlations between single neuron ΔF/F time series reflecting calcium signals (Awake: r = 0.023 ± 0.0003; Anesthesia: r = 0.034 ± 0.0003; WMPSR test: p < 10^–10^; n = 26350 cell pairs; Fig. 2A). In accordance with Greenberg et al. [8], Golshani et al. [18] and Ch’ng and Reid [85], the strongest correlations were observed between pairs of neurons that were close to each other in cortical space, and the strength of pairwise correlations was reduced with increasing distance between neurons (Spatial distance × pairwise correlation; Awake: r = -0.061, p < 10^–10^; Anesthesia: r = -0.073, p < 10^–10^; n = 26350; Fig. 2B). When correlation strength was studied as a function of the similarity in preferred direction under anesthesia, we found a fairly weak, but significant relationship (Fig. 2C), whereas this effect was smaller in awake mice (Direction difference × pairwise correlation; Awake: r = -0.07, p = 0.04; Anesthesia: r = -0.09, p = 0.008; n = 846). The observed relationship under anesthesia confirms earlier findings [73,85–87], whereas the weak relationship under wakefulness has not been reported before.

Recordings in awake animals suffer from increased in- and out-of-plane movement artifacts related to animal motion. Based on video tracking in 4 (out of 10) recordings, we calculated that animal movement occurred in less than 5% of the total length of those recordings. The corresponding movement artifacts will mostly lead to sudden synchronized drops in fluorescence within ROIs. This is because cells that, as a consequence of movement, briefly appear in the image plane, are unlikely to have been defined as ROI’s and therefore the opposite effect (a sudden increase in fluorescence) will less likely occur. Out-of-plane movement artifacts will therefore lead to cellular calcium activity to appear more strongly synchronized, which could result in a difference in correlations between the awake and anesthetized state. Our data, however, shows the opposite, a reduction in correlations between calcium imaged time series in recordings from awake mice. Moreover, if the drops and increases in fluorescence were both occurring at the same rate, this would lead to increased variance of correlations in the awake state, which we did not observe. Therefore out-of-plane movement is unlikely to account for the above results on correlations in activity patterns.

Under anesthesia, the neocortex typically displays slow oscillations in the local field potential, to which many neurons show phase-locked firing of action potentials [7]. The fluorescence signal of the neuropil, the area between cell bodies and blood vessels, has been shown to be correlated to the local field potential and is thought to reflect generalized synaptic inputs to the imaged region [72]. Indeed, the power spectrum of the time series of the neuropil region showed stronger low-frequency activity (0.5–2.0 Hz) in the anesthetized condition as compared to the awake condition (Fig. 2D), which was confirmed by a significant main effect of anesthesia (ANOVA, F_(1,702)_ = 30.23, p < 10^–7^; n = 10 sessions) and a significant interaction effect between anesthesia and frequency (ANOVA, F_(38,702)_ = 2.07, p = 0.0002; n = 10).

In contrast to neuropil signals, cellular ΔF/F time series did not reveal a significant anesthesia-induced increase in power across the low-frequency domain (0.5–2.0 Hz; Main effect anesthesia: ANOVA, F_(1,53508)_ = 0.9, p = 0.34; Interaction effect anesthesia-frequency: ANOVA, F_(38,53508)_ = 0.83, p = 0.76; Both n = 687 neurons; Fig. 2E). Nonetheless, the cross spectrum coherence between simultaneously recorded cellular and neuropil signals was increased for the lower frequencies (0.5–2.0 Hz; Main effect anesthesia: F_(1,53508)_ = 358.5, p < 10^–10^; Interaction effect anesthesia-frequency: ANOVA, F_(38,53508)_ = 13.57, p = 0.0002; n = 687 neurons; Fig. 2F). This suggests a stronger modulation of single neuron calcium activity by common input (as reflected in the neuropil signal) under anesthesia.

Cross-spectrum coherence analysis across all simultaneously recorded cells revealed that single-neuron calcium activity time series correlated most strongly in the low-frequency range (0.2–0.5 Hz) under anesthesia (Main effect of experimental conditions: ANOVA, F_(1,2055222)_ = 237.3, p < 10^–10^; Interaction effect anesthesia-frequency: ANOVA, F_(38,2055222)_ = 33.01, p < 10^–7^; both n = 26350 cell pairs; Fig. 2G), which is potentially a consequence of multiple neurons entraining their activity to a single slow rhythm (< 1 Hz) that is more prominently present under anesthesia (see Fig. 2D; [7,88]). Coherence of cellular calcium time series in the range of 0.15–0.25 Hz did not differ between the awake and anesthetized state, while cellular-neuropil coherence did differ in this range. This may reflect a difference in how anesthesia influences combined cortical input signals (neuropil-cellular coherence, mainly depending on synaptic and dendritic activity; Fig. 2E) versus local cortical processes (cellular-cellular coherence; mainly depending on somatic spiking activity; Fig. 2F). These observations confirm that coherence in calcium activity of primary visual cortex neurons is dependent on anesthesia and frequency in a similar fashion as previously described using action potential activity or estimated firing rates [7,8,18].

### Reduced tuning to movement direction under anesthesia

**Fig 3:**
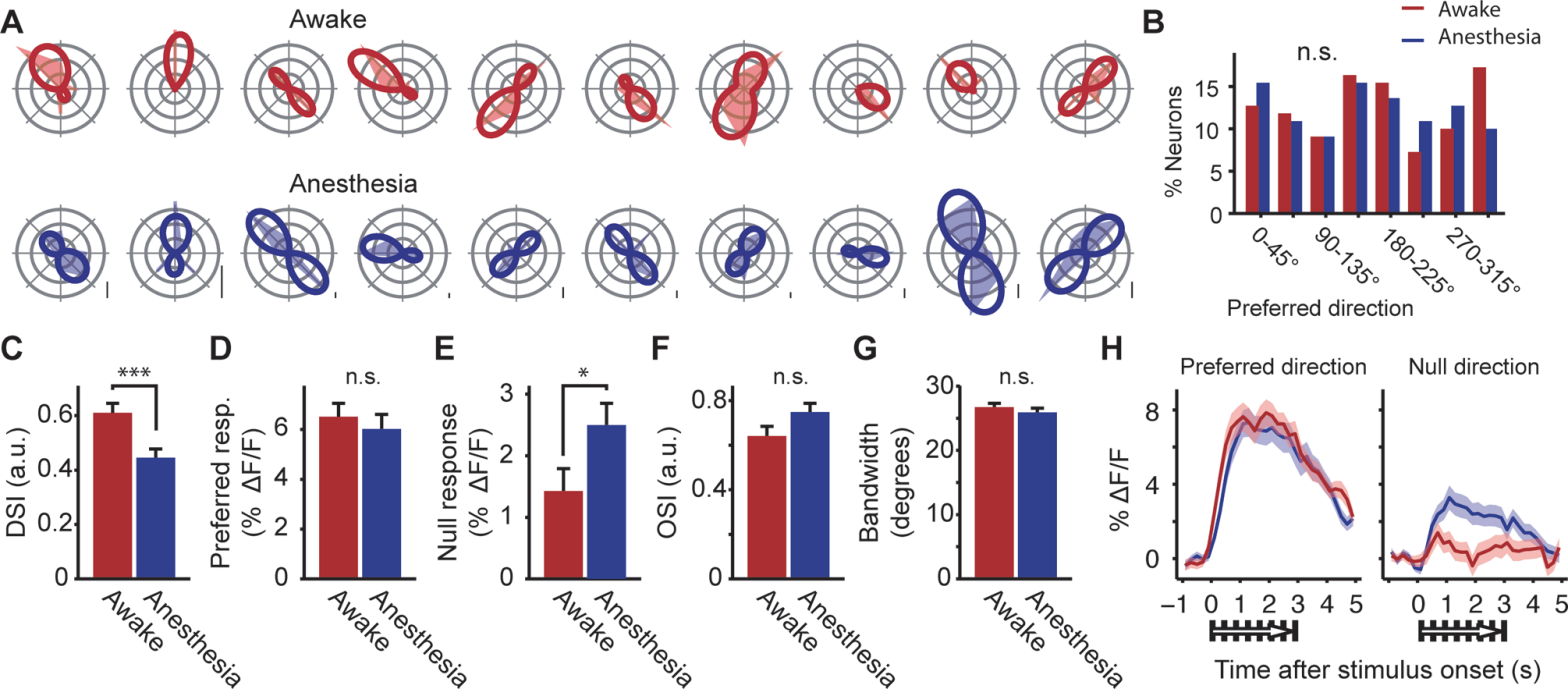
Orientation tuning of visual cortex neurons. (A) Top row: Polar plots show raw tuning curves and curve fits (dotted lines) of significantly orientation-tuned neurons in the awake condition. Bottom row: Idem, but for the same neurons under anesthesia. Scale bar measures 2% ΔF/F. (B) Distributions of preferred directions. No significant differences were found between awake and anesthesia. (C) Mean (± SEM) direction selectivity index (DSI). (D) ΔF/F response to the preferred direction. (E) ΔF/F response to the null direction. (F) Mean orientation selectivity index (OSI). (G) Mean tuning curve bandwidth. (H) Average (± SEM) ΔF/F response to visual stimulation by preferred (left panel) and null direction (right panel) across cells as a function of time around stimulus onset. All panels: Red bars and lines indicate data from the awake condition, blue bars and lines represent data from the anesthetized condition (* p = 0.014, *** p = 0.00017).

Tuning curves were made for 110 neurons that were significantly orientation-tuned across both conditions (Hotellings T^2^ test, p < 0.05). The average stimulus-induced ΔF/F responses in awake and anesthetized recordings were fitted with two-peaked 360° wrapped Gaussian curves separately (Fig. 3A; [76]). Curve fitting was implemented because it significantly reduced variability in estimation of tuning curve parameters under a range of signal to noise ratios (See Materials and Methods; S1 Fig.). The mean squared error of the curve fit was small in both conditions, but significantly smaller for the anesthetized state (Awake = 0.43% ± 0.05% ΔF/F; Anesthesia = 0.19% ± 0.02% ΔF/F; WMPSR test, p < 10^–6^). Many tuning curves recorded under anesthesia correlated significantly with, but were not identical to, tuning curves recorded in the awake condition (mean tuning curve correlation: r = 0.26 ± 0.06; p < 0.05 for 102 out of 110 tuning curves). The overall distribution of preferred directions did not significantly differ between the awake and anesthetized state (Kolmogorov-Smirnov test; D = 0.08, p = 0.84, n = 110; Fig. 3B).

We observed, however, that direction selectivity was greater in awake animals (DSI; Awake: 0.61 ± 0.04; Anesthesia: 0.45 ± 0.03; WMPSR test, p = 0.00017; n = 110; Fig. 3C). This difference could not be explained by a large change in ΔF/F response to the preferred direction (WMPSR test, p = 0.06; n = 110; Fig. 3D), but rather by a strong decrease in ΔF/F response to the movement direction opposite to the preferred direction (i.e. null direction) when neurons were recorded in the awake state (Awake: 1.4% ± 0.36% ΔF/F; Anesthesia: 2.5% ± 0.36% ΔF/F; WMPSR test, p = 0.014; n = 110; Fig. 3E). This decrease in average ΔF/F was apparent in both the ‘early’ (0–1s) and the ‘late’ (1–3s) response to the Null direction (Fig. 3H). The difference in direction selectivity index did not result from a general increase in tuning curve selectivity in awake animals, because bandwidth and orientation selectivity (OSI) did not significantly differ between the awake and anesthetized state (WMPSR test, Bandwidth: p = 0.18, OSI: p = 0.06; n = 110; Fig. 3F and 3G; see also [51]).

It can be argued that, even though the order of recording the awake and anesthesia conditions was counterbalanced across experiments, the reduction of the response to the null direction could result from an interaction between recording duration and awake/anesthetized state. To examine the possibility that the observed differences were the result of a recording-sequence effect, we tested for the same effects in a control dataset including only tuning curves from the first recorded condition of each experiment. Although the comparison was now done between different groups of cells, direction selectivity was still significantly greater and the response to the null direction was still significantly reduced in the awake condition as compared to anesthesia (Mann-Whitney U test, DSI: p = 0.007, null response: p = 8.4·10^–5^; Awake first: n = 79, Anesthesia first: n = 31).

In the awake condition, mice occasionally displayed body movement on the walking belt (Fig. 1A, red line). Niell and Stryker [51] reported that visually evoked firing-rate responses are much larger in amplitude when an animal displays active running behavior as compared to when it is not. Although in our experiments the mice were mostly sitting still on the walking belt (movement was only detected in 4.2% ± 1.2%, mean ± SD, of the total experiment), it is important to investigate whether increased visual responses during episodes of movement influenced estimates of direction selectivity. Sufficient information on rotational movement of the walking belt was available in 4 recording sessions. Because the awake condition was recorded before the anesthesia condition in each of these sessions, it was not possible to directly compare awake/anesthesia effects in the same cells, while excluding a sequence bias. Therefore, we first investigated how direction selectivity is affected by moving/non-moving episodes in the same cells, while not comparing these episodes to the anesthetized condition. For cells recorded in these sessions, tuning curves were calculated using data from trials in which the animal was moving and not moving separately. The ΔF/F responses to the preferred directions of the recorded neurons were larger in tuning curves measured during movement (12.7% ± 1.3%) as compared to those in tuning curves from the same neurons, measured when the animal was immobile (7.5% ± 0.7%; WMPSR test, p = 3.8 · 10^–5^; n = 70). Responses to the null direction of those tuning curves, however, also increased in magnitude when measured during body movement and the resulting direction selectivity did not change significantly (DSI; Moving: 0.61 ± 0.04; Not moving: 0.56 ± 0.04; WMPSR test, p = 0.31; n = 70). Second, we compared the direction selectivity of cells recorded during the non-movement periods (all from recordings with the awake measurement first) to cells from recordings with the anesthesia measurement before the awake measurement. In this case there will be no sequence bias, but it will not be the same cells being compared. Still, the average direction selectivity index was significantly higher in the data from awake animals compared to the anesthetized ones (DSI; Not moving-awake: 0.56 ± 0.04, N = 70; Anesthesia: 0.36 ± 0.06, N = 31; Mann-Whitney U test, p = 0.023). Together with the low prevalence of movement episodes, this makes it unlikely that the awake-anesthesia difference in direction selectivity was the result of altered cortical processing during walking/running.

Two-photon imaging in awake animals must be scrutinized for movement artifacts. In-plane (X-Y) movement was corrected using offline image registration (see Methods) and recordings with strong out-of-plane movement (i.e., in the Z-dimension) were rejected. Still, it cannot be fully excluded that increased movement artifacts explain increased direction selectivity in awake animals. Because movement in the Z-dimension virtually always occurs together with in-plane movement, we used X-Y movement to indicate potential periods of out-of plane (Z) movement. Next, individual trials in which the maximum instantaneous XY shift of the uncorrected image time series during the baseline or stimulus period exceeded 2 pixels, which spanned on average 1.6 μm but varied slightly from session to session, were removed from the dataset. The direction selectivity index, now only based only on trials in which no movement in the X-Y plane was observed, still differed significantly between the awake- and anesthesia condition (DSI; Awake: 0.64 ± 0.05; Anesthesia: 0.49 ± 0.04; WMPSR test, p = 0.015; n = 70), which indicates that in- and out-of-plane movement artifacts cannot explain the stronger direction-selectivity that was observed in awake mice.

Even in a head-restrained preparation, mice can make eye movements. Optokinetic eye movements (following the motion direction of drifting bars), however, are virtually absent at the drifting grating speed of 2 cycles/second that was used in the present study [89]. Nonetheless, spontaneous saccade-like eye movements, which occur independently of presented stimulus orientation, might affect direction selectivity along the axis of those eye movements. Because these spontaneous eye movements occur mostly along the horizontal movement axis [90], we calculated the direction selectivity index for cells with a preferred direction along each axis of movement separately (S2 Fig.). If the presence of spontaneous eye movements in the awake condition would explain the increased direction selectivity, this effect should be strongest for the movement axis of these eye movements (horizontal). Direction selectivity was, however, not larger for cells that were tuned to movement along the horizontal axis (90 and 270 degrees). Therefore, spontaneous eye movements are unlikely to explain the difference in direction selectivity that was observed between awake and anesthetized animals.

The decrement in direction selectivity may be indicative of a more general reduction of response selectivity for visual stimuli under anesthesia. To control for this, we calculated sparseness of single neuron and population responses. Sparseness measures are appropriate to address the question of generalized loss of response selectivity, as they do not assume significant orientation or direction tuning. Sparseness of average single neuron and population responses was quantified using a measure that was made appropriate for responses ranging from −∞ to ∞ (see Materials and Methods; [80,81]). Neither single-neuron response sparseness nor sparseness of the population response to visual stimuli differed significantly between the awake and anesthetized state (WMPSR test, Single neuron: p = 0.24; n = 110, Population response: p = 0.63; n = 10). At first glance, these results seem incompatible with the anesthesia-induced reduction in direction selectivity. Going back to Fig. 3F however, orientation selectivity was slightly higher (although non-significant) in the anesthetized condition and likely compensated the sparseness measure for the loss of directional selectivity.

Anesthesia significantly altered dynamics of neuropil signals during periods of spontaneous activity (Fig. 2D). Although we corrected for neuropil-contamination, we examined the possibility that the difference in direction selectivity was due to residual (non-corrected) neuropil fluorescence in cellular signals, or alternatively was a result of the correction method itself. We thus defined direction selectivity of significantly orientation-tuned neurons, but based on NON-neuropil-corrected calcium imaging data. If the difference in direction selectivity originated from the neuropil signal, this difference should be stronger in uncorrected data, while if the difference in selectivity resulted from the correction method, the effect should be smaller in uncorrected data. Overall, the direction selectivity index was a bit lower in data that was not corrected for neuropil contamination, but the effect size between the awake and anesthetized state remained the same (Awake: 0.54 ± 0.04; Anesthesia: 0.41 ± 0.04; WMPSR test, p = 0.022; n = 71).

Next, we plotted the neuropil signal in response to visual stimulation for both states (awake/anesthetized) and the eight presented movement directions separately (S3A Fig.). This revealed that the visually evoked temporal dynamics of neuropil signals did not clearly differ between states. In addition, the neuropil signal did not seem to be systematically tuned to a single movement direction that was the same across all recordings (S3B Fig.), but the neuropil tuning curve of each separate recording did show a peak that became visible when neuropil tuning curves were aligned to individual ‘preferred directions’ (S3C Fig.). This peak may reflect a true directional imbalance in the strength of the neuropil response, or might be merely a consequence of random activity. Neuronal ΔF/F responses to the respective ‘preferred direction’ of the neuropil signal were slightly larger in the awake condition (S3E Fig.). This could be caused by increased neuropil contamination, or alternatively by cellular signals that co-occurred with the neuropil signal (but are not optical contamination). This signal may have influenced estimates of direction selectivity for cells tuned to this direction. The signal, however, cannot affect neurons with a preferred direction different from the neuropil peak, as the DSI is quantified using only the preferred and null response. The direction selectivity of the subset of cells with preferred orientations (Preferred direction and Null direction) away from the neuropil peak was still significantly larger in awake compared to anesthetized mice (DSI; Awake: 0.44 ± 0.04; Anesthesia: 0.31 ± 0.03; WMPSR test, p = 0.0002; n = 72; S3F Fig.). Therefore, although neuropil signals differ somewhat between the awake and anesthetized state (spontaneous activity: Fig. 2D; visual stimulation: S3 Fig.), they do not provide an alternative explanation for the difference in neuronal direction selectivity.

**Fig 4:**
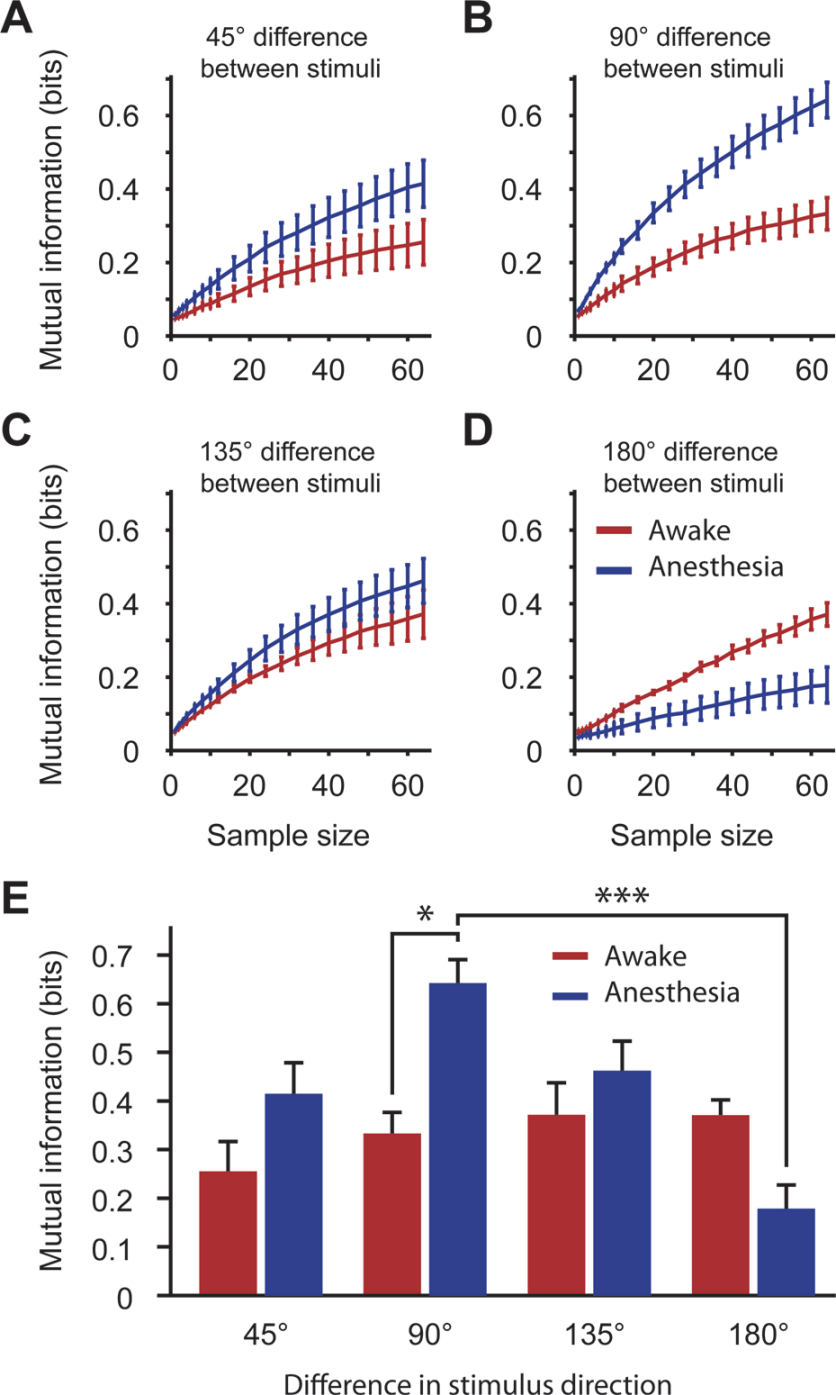
Population coding: Mutual information of stimulus orientation and direction as a function of ensemble size. (A) Mutual information of single trial ΔF/F responses to stimuli that differed 45° in movement direction. Mutual information was quantified as mean bits per trial across differently sized groups of neurons (ranging from 1 to 64 cells), averaged (± SEM) across unique stimulus combinations for the awake (red line) and anesthetized (blue line) condition. (B) Idem, for stimuli differing 90° in direction. (C) Idem, for stimuli differing 135°. (D) Idem, for stimuli differing 180° (opposite movement directions). (E) The mean mutual information on stimulus direction for a sample size of 64 neurons. Mutual information for each unique stimulus combination was determined by averaging across 2000 unique samples of 64 neurons from the entire population. The mean (± SEM) was taken across unique stimulus combinations (n = 8 for 45°, 90° and 135°; n = 4 for 180°). Data from awake (red) and anesthetized (blue) mice are displayed separately as a function of the angular difference in stimulus combinations (* p < 0.05, *** p < 0.001).

Depending on recording conditions, direction selectivity may be highly sensitive to noise in tuning curves: A spurious large-amplitude response may create the apparent notion of direction selectivity in a neuron that is merely orientation-tuned. Because the awake state is associated with more sources of stimulus independent fluctuations in calcium signals than the anesthetized state (e.g. movement artifacts, eye movements), we quantified to what extent the difference in direction selectivity is dependent on the signal-to-noise ratio of the cells included in our analysis. Signal-to-noise ratio was quantified by dividing the mean response to the preferred direction by the standard deviation of the individual trial responses for this direction. Subsequently, we calculated tuning curve parameters for the entire group of cells while, in each iteration, removing the cell with the lowest signal-to-noise ratio from the remaining sample. This resulted in a curve that displays the difference between the awake and anesthetized state for increasing signal-to-noise ratios of the included group of cells (S4 Fig.). If the larger direction selectivity index in the awake condition would be explained by increased noise, this difference should be reduced with each removal of a low signal-to-noise cell from the group, until no difference in direction selectivity index would be visible when considering the group containing only the highest signal-to-noise cells. As expected, the response amplitude to the preferred- and null direction increased with rising signal-to-noise ratio, which resulted in increasing values for the orientation selectivity index, depending on which data were used for the SNR quantification (i.e., an increasing OSI under anesthesia when cells were selected on SNR in the same condition, and similarly for the awake condition; S4B-D and G-I Fig.). Tuning width and direction selectivity index, however, remained largely unchanged (Fig. 4A,E,F and J), which indicates that the awake-anesthesia difference in direction selectivity index is not due to increased noise (or reduced SNR) in the awake condition.

Direction selectivity is defined as a feature of an orientation-tuned neuron. Our inclusion criterion for orientation tuning was determined by a circular test (Hotelling’s T^2^ test), which indicates whether a vectorial sum of responses across all orientations or directions is significantly different from zero. One may argue that the difference in DSI depends on a lesser orientation-tuned subset of cells, which would render this result less meaningful. In a final control analysis, we therefore excluded all cells that had an OSI lower than 0.5 in either the awake-, anesthetized, or both recording phases. In the resulting high OSI-dataset, the DSI was significantly different between the anesthetized and awake state (DSI; Awake: 0.66 ± 0.05; Anesthesia: 0.47 ± 0.05; WMPSR test, p = 0.002; n = 57).

Effects of anesthetics can vary with weight and age of an animal. We used only adult male mice, but their ages and weights varied considerably. To exclude that the effects of anesthesia on DSI were carried by a large number of neurons recorded from only a few animals, we calculated a mean DSI per animal. Analysis across animals showed the same anesthesia dependent difference in direction selectivity (DSI; Awake: 0.66 ± 0.05; Anesthesia: 0.48 ± 0.07; WMPSR test, p = 0.016; n = 9 mice). We next considered if weight and age of the mice correlated with the awake/anesthesia difference in DSI (ΔDSI). Weight (24.7 to 33.3 gram, mean (±SD): 27.4 ± 3.1 gram) nor Age (57 to 134 days, mean (±SD) 99 ± 22 days) correlated significantly with this measure (Pearson correlation coefficient; Weight-ΔDSI: r = 0.63, p = 0.09; Age-ΔDSI: r = 0.18, p = 0.68; n = 8, one mouse is missing because weight/age data could not be retrieved). Although there seemed to be a trend towards heavier mice having a stronger anesthesia induced reduction in DSI, the low number of animals in this analysis makes it hard to interpret.

Together, these results show that anesthesia does not significantly change single-cell orientation tuning while it reduces direction tuning. Although the observed changes may be specific to the combination of isoflurane anesthesia with buprenorphine analgesia, they cannot be explained by a general loss of selectivity, differences in recording order, state-dependent (moving versus quiet) changes in visually evoked response amplitudes, Z-motion artifacts, eye movements, altered neuropil dynamics or noise on tuning curves. The change in direction selectivity of tuned cells is therefore likely a direct consequence of anesthetic action in the central nervous system.

### Population estimates of mutual information on stimulus direction are reduced under anesthesia

Higher cortical areas may read out stimulus orientation and direction information from V1 population activity using a linear decoding principle [91]. To test whether anesthesia-induced alterations in tuning parameters and correlations in V1 population activity impaired coding of stimulus orientation or direction in larger groups of neurons, we implemented such a decoding algorithm and calculated the mutual information on stimulus orientation and direction on its output. Using Euclidean distance metrics on population vectors of single trial ΔF/F responses, the decoding algorithm estimated correct stimulus identity for pairs of trials that differed in movement direction to a variable degree (see Materials and Methods). With a 45° sampling resolution of stimulus direction, the stimulus protocol allowed to distinguish eight pairs with an orientation difference of 45°, eight pairs differing 90° and eight pairs differing 135°. Additionally, four pairs had the same stimulus orientation and differed only in movement direction. Mutual information was calculated on the output of the decoding algorithm for populations of 1 to 64 neurons using the Panzeri-Treves toolbox [83]. The estimates of mutual information were corrected for bias due to a low number of trial repetitions using bootstrap subtraction; it should be noted that this method can lead to an overall underestimation of mutual information (see Materials and Methods).

Mutual information increased with larger populations of cells in all of the conditions (Fig. 4A-D). Focusing on the estimate for groups of 64 neurons, a two way Anova test revealed a significant main effect for wakefulness versus anesthesia (ANOVA, F_(1,48)_ = 4.43, p = 0.041), a main effect for difference in stimulus direction (ANOVA, F_(3,48)_ = 4.36, p = 0.009) and a significant interaction between both variables (ANOVA, F_(3,48)_ = 4.73, p = 0.006; Mutual information in bits for: 45°, Aw: 0.26 ± 0.06, An: 0.41 ± 0.06; 90°, Aw: 0.33 ± 0.04, An: 0.64 ± 0.05; 135°, Aw: 0.37 ± 0.07, An: 0.46 ± 0.06; 180°, Aw: 0.37 ± 0.03, An: 0.18 ± 0.05; Fig. 4E). Specifically, under anesthesia, mutual information for differences in stimulus orientation (90° difference) was significantly higher than for differences in stimulus direction (180° difference; p < 0.001 after multiple comparisons), but also compared to stimulus orientation differences in the awake state (p < 0.05). In awake animals, mutual information on stimulus orientation and direction was similar for every orientation / direction difference (45°, 90°, 135° and 180° difference, all p > 0.70). The observation of an anesthesia-dependent reduction in mutual information of stimulus direction is consistent with the observation of reduced direction selectivity in single neurons (Fig. 3C). It demonstrates that the anesthesia-related changes are strong enough to hamper population processing of movement directions across large groups of V1 neurons, and are therefore likely to influence the propagation of this information to downstream visual areas.

### Direction selectivity and spontaneous correlation patterns do not covary in a single subset of neurons

We hypothesized that the decreased direction selectivity may rely on the same set of neural mechanisms underlying the increase in spontaneous activity correlations under anesthesia. Under this hypothesis, a correlation between the two phenomena is expected. First we tested whether there was a significant statistical relationship between direction selectivity and pairwise activity correlations either in the awake or anesthetized state by calculating Pearson’s correlation coefficient between the average direction selectivity index of a pair of neurons and the correlation between their ΔF/F time series (either spontaneous or evoked activity). These coefficients were normalized per session to prevent bias from between-session differences. No such correlation was found in data from awake or anesthetized mice for correlations during evoked activity and DSI (Correlation visual stimulation × DSI, Awake: r = -0.024, p = 0.49; Anesthesia: r = -0.001, p = 0.97; n = 846). Secondly, pairs of neurons that displayed the most pronounced increment in correlations (either during spontaneous or evoked activity) did not systematically show a greater decrease in direction selectivity under anesthesia (ΔCorr. spont. act. × ΔDI: r = 0.0049, p = 0.89; ΔCorr. vis. stim. × ΔDI: r = 0.045, p = 0.19; n = 846). Incidentally, we found that pairs of neurons with broader bandwidths showed stronger positive correlations in calcium activity patterns during visual stimulation (ΔCorr. vis. stim. × Δbandwidth: r = 0.11, p = 0.001; n = 846), which is likely caused by an increased chance on overlap of the tuning curves resulting in signal correlations, because no such effect was seen for correlations measured during spontaneous activity. These results show that anesthesia-related changes in tuning of visual cortex neurons and correlations in their spontaneous activity do not covary in the same subset of neurons and may therefore be caused by two independent mechanisms operating in parallel. However, the relation between changes in tuning and correlations during periods of visual stimulation deserves further scrutiny.

### Anesthesia-induced changes in tuning and correlations are related to changes in mutual information of stimulus orientation and direction

Changes in single-neuron tuning curve parameters and pairwise correlations (Fig. 2A) between the awake and anesthetized state may interact in how they affect population coding of stimulus orientation and direction. To understand the individual contributions of these parameters to orientation and direction coding, we first determined the mutual information on stimulus orientation and direction for the activity patterns of all possible simultaneously recorded triplets of orientation-tuned neurons in our dataset (n = 5275) and implemented a linear regression analysis, which estimated independent predictive strength of variation in tuning and correlation structure between the awake and anesthetized state on the mutual information conveyed by those neurons. This approach exploits the stochastic variability in changes from the awake to anesthetized state for all measured parameters of individual triplets of neurons to assess their independent influences on population coding and without requiring overall anesthesia-related changes in those parameters (differences in overall means). Using this analysis, we can assess which parameters affect population coding, even if there would not be an overall awake/anesthesia effect. As a consequence, parameter-specific results obtained from this analysis do not necessarily reflect overall changes in those parameters between the awake and anesthetized state.

For each triplet, we assessed the parameters mutual information, mean bandwidth, orientation- and direction selectivity index, ΔF/F response to the preferred and null direction and the correlation between pairs of ΔF/F time series during spontaneous activity and visual stimulation and z-scored them per recording-session. The differences in mutual information between the awake and anesthetized state were subsequently fitted by a model assuming linear interactions with the differences in tuning parameters and correlated activity. Resulting beta coefficients describe the relations between changes in each predictive variable and changes in mutual information, with positive values indicating that a positive change (from awake to anesthetized state) in the triplet-average of a parameter correlated positively with an increase in mutual information. For instance, a positive beta weight for +Δ*R*_*pref*_ indicates that an increase of the response to the preferred direction (*R*_*pref*_) correlates positively with an increase in mutual information, or that a decrease in *R*_*pref*_ correlates positively with a decrease in mutual information.

**Fig 5:**
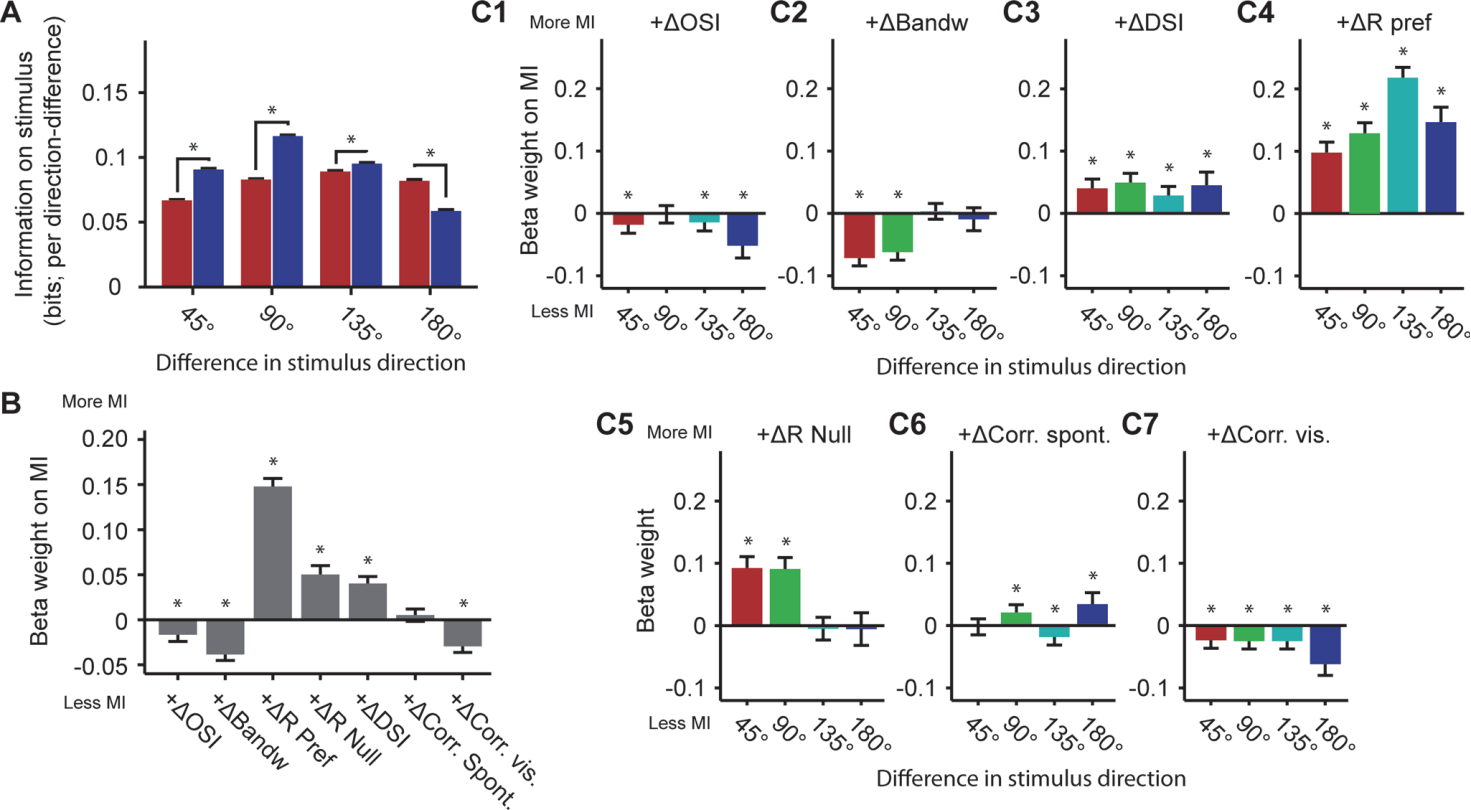
Regression analysis on neuron triplets: Relation between mutual information, tuning properties and correlated activity. Interaction between tuning curve parameters, correlation structure and mutual information for all simultaneously recorded triplets of neurons. (A) Mutual information on stimulus for selected pairs of stimuli differing 45°, 90°, 135° and 180° in direction. This part of the triplet analysis essentially confirms the results from Fig. 4. (B) Regression analysis showing the relations between the normalized (z-scored) anesthesia-induced changes in tuning parameters, correlations in activity patterns, and changes in mutual information on stimulus orientation and direction (across all possible stimulus direction differences) going from the awake to the anesthetized state for all simultaneously recorded triplets of neurons. This graph can be read by translating a negative beta weight for +ΔCorr.vis, for example, as a condition in which anesthesia-induced increments in activity correlations recorded during visual stimulation covary with a decrease in mutual information on stimulus direction in the awake state. (C) Beta weights for predictor variables per difference in stimulus direction. Data are displayed as mean ± 99% confidence bounds (* p < 0.001).

Upon seeking to confirm that MI for specific orientation and direction differences was altered for triplets, as found for large populations (Fig. 4), we indeed found that MI for direction differences computed from triplets of neurons was significantly reduced under anesthesia, while MI on stimulus orientations was increased (WMPSR test; Per direction difference: 45°: p < 10^–10^; 90°: p < 10^–10^; 135°: p < 1.08 · 10^–5^; 180°: p < 10^-10^; n = 5275 in all comparisons; Fig. 5A).

The fitted linear model predicted the observed awake-anesthesia changes in mutual information significantly, but not very strongly (R^2^ = 0.0207, p < 10^–10^; i.e. mutual information was calculated for stimulus differences of 45°, 90°, 135° or 180° separately and then pooled to get an overall estimate). The anesthesia-induced change in strength of ΔF/F responses to preferred and null directions and change in direction selectivity index were the strongest positive predictors in the overall model (Fig. 5B). Whereas anesthesia-induced changes in pairwise correlations during spontaneous activity did not predict mutual information significantly, increases in pairwise correlations under anesthesia during visually evoked activity significantly predicted less mutual information (Fig. 5B). Thus, an increase in redundancy of visually evoked calcium responses (as measured by correlations) may be hypothesized to induce a decrease in mutual information on stimulus identity.

To assess the independent influences of changes in tuning and correlation parameters on awake-anesthesia differences in mutual information on the basis of differences in stimulus orientation and direction, we fitted the linear model to datasets with specific differences in decoded stimulus directions (45°, 90°, 135° or 180°; Fig. 5C1-C7). Independently of OSI (Fig. 5C1), bandwidth (Fig. 5C2) and response amplitude to the preferred (Fig. 5C4) and null directions (Fig. 5C5), correlations during visual stimulation (Fig. 5C7) had a negative impact on population coding of direction differences, while -as expected- direction-selectivity itself had a positive effect (Fig. 5C3).

Besides these clear effects, we noted a few more subtle effects. First, larger bandwidths negatively affected mutual information for stimulus differences of 45° or 90° (Fig. 5C2), which can be explained by the observation that broader tuning curves under these conditions provide less information on orientations with higher similarity. Second, and related to this, the change in response to the null direction provided useful information on stimulus orientation when the ‘to be discriminated’ stimuli were not of opposite direction, but differed 45° or 90° (Fig. 5C5, left bars). Last, an increase in correlations during spontaneous activity under anesthesia positively predicted decoding performance for 90° and 180° differences (Fig. 5C6), which, however, may be related to orientation-tuning dependent correlations (as shown in Fig. 2C below). The main conclusion from the linear model is that anesthesia-induced changes in correlations during visual stimulation and the observed changes in direction selectivity could both underlie part of the anesthesia-dependent decrement in population coding of stimulus directions, but do not fully explain the observed reduction in decoding performance under anesthesia.

### Visual stimulation decorrelates overall calcium activity in anesthetized V1

**Fig 6:**
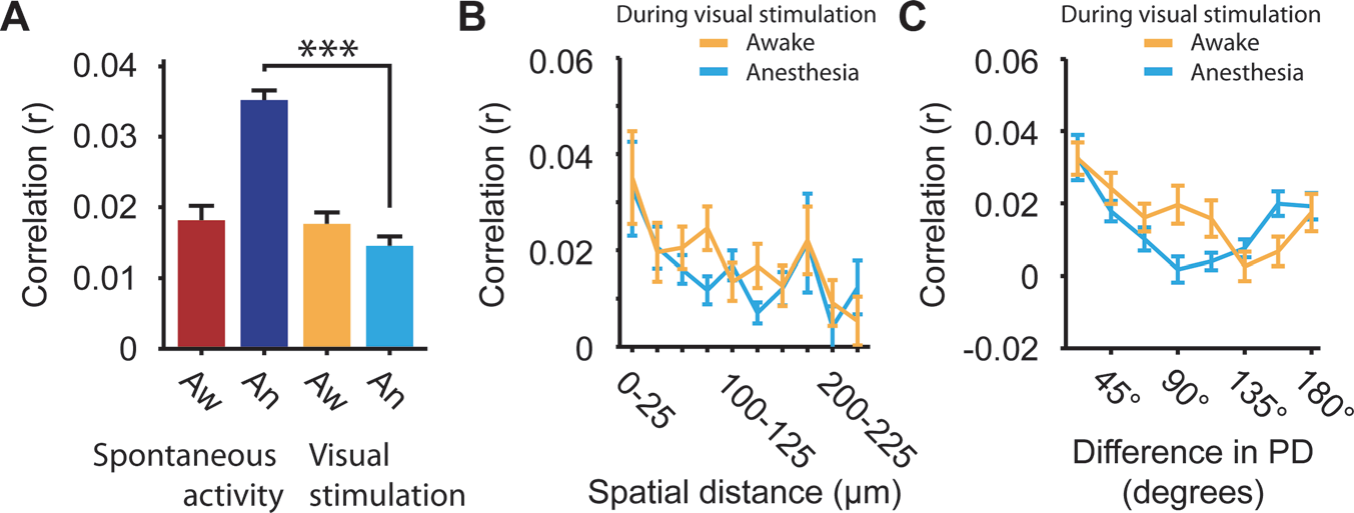
Pairwise correlations in cellular calcium signals during visual stimulation. (A) Correlation between calcium time series of all simultaneously recorded pairs of orientation tuned neurons during spontaneous activity or visual stimulation, studied under awake (Aw) and anesthetized (An) conditions (*** p < 10^–10^). (B) Pairwise correlations during visual stimulation as a function of spatial distance between cell bodies of orientation tuned neurons (Yellow: Awake, r = -0.10, p = 0.0026; Blue: Anesthesia, r = -0.077, p = 0.026; n = 846). (C) Pairwise correlations during visual stimulation as a function of the difference in preferred direction of orientation tuned neurons (Yellow: Awake, r = -0.14, p = 4.0 * 10^–5^; Blue: Anesthesia, r = -0.075, p < 0.028; n = 846).

We observed an inverse relationship between correlations during visual stimulation and decoding of population activity, especially for opposite stimulus directions (Fig. 5C7), which may be taken to suggest that increased redundancy, as gauged by pairwise correlations, could at least partly underlie the reduction in direction coding. Surprisingly, the data do not support this view. Although under anesthesia neurons are more responsive to the null direction (Fig. 3E) and showed higher correlations in spontaneous activity (Fig. 2), overall pairwise correlations in calcium activity of orientation-tuned neurons did not significantly differ between the awake and anesthetized state during visual stimulation (WMPSR test, p = 0.092; n = 846; Fig. 6A). Rather, anesthesia revealed a significant decorrelation when comparing calcium activity during visual stimulation with spontaneous calcium activity (WMPSR test, p < 10^–10^; n = 846; Fig. 6A, dark blue and cyan bar). In awake animals, no such decline was observed (WMPSR test, P = 0.94; n = 846), which suggests that the cortical network was already in a decorrelated state during spontaneous activity.

Given the strong decorrelating effect of visual stimulation under anesthesia, we next asked whether visual stimulation under anesthesia abolishes structured spatial patterning of correlations altogether. This was not the case. Despite the general reduction of correlation strength by visual stimulation under anesthesia, correlations still diminished with increasing spatial distance (Spatial distance × pairwise correlation; Visual-stim; Awake: r = -0.10, p = 0.0026; Anesthesia: r = -0.077, p = 0.026; n = 846; Fig. 6B). We also examined whether the dependence of correlations on tuning similarity (Fig. 2C) was still intact under visual stimulation conditions. Pairs of neurons with similar preferred directions were more strongly correlated in their ΔF/F time series recorded during the period of visual stimulation in both the awake and anesthetized condition as compared to dissimilar pairs (Direction difference × pairwise correlation; Awake: r = -0.14, p = 4.0 * 10^–5^; Anesthesia: r = -0.075, p < 0.028; n = 846; Fig. 6C).

In summary, visual stimulation lead to a decorrelation of overall calcium activity patterns in anesthetized cortex, to a level comparable to the awake situation. This argues against a causal effect of the increased correlations between spontaneous activity patterns as being detrimental to population coding for stimulus direction and suggests that anesthesia-induced changes in decoding performance should be ascribed to the observed changes in tuning curve selectivity. Under anesthesia, the overall decrement in correlations induced by visual stimulation occurs in the continued presence of structural relationships between correlations, spatial distance and tuning similarity between neurons.

## Discussion

Using in vivo two-photon calcium imaging we recorded spontaneous and evoked activity patterns in populations of orientation-tuned neurons studied during the awake and anesthetized state in the same experiment. As in previous studies, pairwise correlations in spontaneous calcium activity were stronger under anesthesia and dependent on spatial distance and similarity in orientation tuning. At the single cell level, orientation selectivity remained unaffected by anesthesia while direction selectivity was reduced in anesthetized mice, associated with an increased response to the null direction. Consistent with this, mutual information for stimulus direction was reduced under anesthesia (Fig. 4), even when estimated for large populations of neurons, and a linear regression-based approach indicated that anesthesia-induced changes in population coding of stimulus direction covaried with tuning curve parameters and changes in correlation patterns under spontaneous and visually stimulated conditions. In the anesthetized recordings, however, visual stimulation led to a decorrelation of calcium activity patterns, to a degree that rendered correlated activity comparable to levels in awake cortex, precluding an effect of correlations to causally underlie the reduction in direction coding and prompting to ascribe it to the observed alterations in tuning curve parameters.

### Methodological considerations

In this study we did not make use of high-resolution eye-tracking. Although rodents can make voluntary eye movements, these are mainly driven by the vestibulo-ocular reflex, which is abolished under stable head-restraint [92]. Second, under head-restraint, most spontaneous saccades are smaller than 20 degrees [43,90,93,94], which is less than one third of the extent of the monitor that was used for visual stimulation. Third, Keller et al. [69] showed that most of the saccades that occur when an animal is head-restrained, happen during running. These saccades, however, were small in amplitude and resulted in little Ca^2+^ activity in mouse visual cortex. Keller et al. suggest that this can be explained by their use of full-field gratings (as in the present study), which results in nearly identical visual stimulation across much of the visual field. Together, these studies suggest that the effects of eye movements in our study, using a head-restraint preparation and a mostly immobile mouse, are unlikely to have exerted a major influence on the experimental results.

An eye movement during viewing of a gray screen could lead to simultaneous activity of cells receiving visual inputs from a region near the border of the screen. Given the correlation in receptive field locations of cells in an imaging plane in mouse V1 [66], this would lead to increased correlations in the condition which has more eye movements; the awake condition. In contrast, correlations were reduced in the awake condition and eye movements consequently do not provide a likely explanation for the observed changes in spontaneous activity patterns between the awake and anesthetized condition.

Still, eye movements during visual stimulation might alter stimulus processing and affect orientation tuning. Indeed, mice can make optokinetic eye movements during presentation of drifting gratings, which renders the projection of smooth movement of a grating jerky on the retina. By consequence, the effective drift frequency of the moving grating would decrease during stimulus-following and increase in swing-back periods. The drift frequencies at which mice perform these optokinetic behaviors, however, are below 1 Hz, while the drift frequency used in this study was 2 Hz, at which optokinetic eye movements are virtually absent [89]. On the other hand, spontaneous eye movements independent of stimulus orientation occur mostly along the horizontal movement axis [90]. Because we did not observe an obvious exaggeration of DSI along horizontal directions specifically (S2 Fig.), rapid spontaneous eye movements do not likely explain the difference in direction selectivity. In summary, the evidence and arguments presented above strongly suggest that the differences between the awake and anesthetized state are not solely the consequence of differences in eye movements between the two states.

Recordings in awake animals may suffer from increased in- and out-of-plane movement artifacts, which leads to sudden synchronized changes in fluorescence and can result in more strongly synchronized calcium activity. Our data, however, shows effects on correlations that cannot be explained by these synchronized changes (see Results). Moreover, control analyses showed that data, with movement artifacts removed, still differed in direction selectivity between the awake and anesthetized state. Therefore potential increased out-of-plane movement is unlikely to underlie the difference in DSI between the awake and anesthetized state.

### Anesthesia affects tuning to visual stimuli

Differences in coding for visual features between awake and anesthetized animals have been studied before, but orientation tuning has generally been thought to remain unaffected by anesthesia [45,46,49,51,95]. Direction selectivity, however, has been reported to be more sensitive to anesthesia [96,97], although no study has, to our knowledge, directly compared parameters describing tuning for movement direction of identical populations of neurons measured in awake and anesthetized animals. The reported changes in direction coding may be dependent on depth, type and method of anesthesia. Effects of anesthetics and analgesics are difficult to compare between studies because there is no standard protocol and different anesthetics such as isoflurane (e.g. [18,54,98]), urethane (e.g. [18,51,67]), ketamine/xylazine (e.g. [8,67]), halothane (e.g. [55]) and others are widely used in visual cortex research. On the other hand, many share commonalities in their molecular function: a potentiation of GABA_A_-induced chloride currents and an inhibition of excitatory currents, mediated by NMDA and AMPA receptors [1,11,12], as well as similarities in network function [7,15]. Moreover, despite the fact that we used different anesthetics compared to some earlier reports on anesthesia-induced changes in correlations and synchrony of V1 activity patterns, the effects reported earlier were reproduced here (Fig. 2; [7,8,18]). Nonetheless, the results obtained using isoflurane in this study cannot be extrapolated to all other forms of anesthesia, but rather illustrate how anesthesia-induced loss of consciousness is associated with changes in cortical function.

Anesthesia attenuates neuronal responses to moving stimuli [39,40]. When comparing orientation-tuning of the same neurons under different levels of halothane / N_2_O anesthesia in the cat, Ikeda and Wright [39] found that preferred orientation did not change, while tuning curves for movement orientation became less sharp as a consequence of reduced response amplitude to the preferred orientation. Niell and Stryker [51], on the other hand, did not find differences in orientation selectivity between sets of neurons recorded under anesthesia and in awake mice. Tuning for pattern-motion direction, as observed in monkey primary visual cortex and area MT, was reduced by anesthesia [55,95], but these effects could be potentially explained from an overall reduction in sensitivity to visual stimuli of cortical neurons [99,100].

Concerning direction tuning, most previous studies reported estimates in awake or anesthetized monkeys and cats to fall within a similar range of values [45,46,51,55,95,99], although Mandl et al. [96] described that direction-selective responses are more variable under anesthesia. Comparison of two recent studies on mouse visual cortex, each focusing on either the awake or anesthetized state but not in the same mice, suggests indeed a difference in tuning for direction, but not orientation, between the awake and anesthetized state (Andermann et al. [53]: Awake mice, mean OSI = 0.54, mean DSI = 0.45; Marshel et al. [98]: Anesthetized mice, 0.8% Isoflurane and Chlorprothixene, mean OSI = 0.50, mean DSI = 0.26; see also [101]). This may be attributable to several methodological factors distinguishing these two studies, but adds indirect support for an anesthesia-induced reduction in motion selectivity, as demonstrated in the current study.

### Visual stimulation decorrelates activity patterns under anesthesia

The entrainment of neuronal spontaneous activity to a slow oscillation in the local field potential and increased correlations between activity patterns of single neurons are pronounced signatures of the anesthetized brain [7,8,102]. We expected that correlations between activity patterns during periods of visual stimulation would be increased under anesthesia as they were for spontaneous activity. Because, under certain circumstances, correlated activity patterns can be detrimental to population coding, this might have helped explain the difference in population coding of direction between the two states (Fig. 4; [19,21]). Although increased correlations between activity patterns during visual stimulation were indeed a negative predictor for reduced mutual information on stimulus direction (as shown by regression analysis; Fig. 5C7), we observed that visual stimulation resulted in a decorrelation of activity patterns under anesthesia, comparable to the level seen in awake mice (during either spontaneous activity or visual stimulation). It is therefore unlikely that reduced information on movement direction resulted from an anesthesia-induced change in activity correlations as recorded during visual stimulation.

The decorrelation of cortical activity may have been induced in a way similar to that observed in vitro, where it was found that periods of self-sustained activity can be either initiated or terminated by activating local intra-cortical or thalamo-cortical afferents [103]. In our study, visual stimulation may have supplied these excitatory inputs to V1 and thus have ‘broken’ the ongoing strongly correlated spontaneous activity patterns. In the intact animal, visual stimulation drives inhibition and excitation almost simultaneously and although inhibition is stronger in the awake as compared to anesthetized state [42], inhibitory inputs may still contribute to decorrelating cortical activity patterns as compared to ongoing spontaneous activity. The negative relation between changes in correlations during visual stimulation and mutual information (Fig. 5C7) may therefore be alternatively explained by visual stimulation providing the strongest external drive to the most visually responsive neurons, inducing the strongest reduction in correlative activity in this subset that conveys most mutual information on the stimuli.

### Potential mechanisms underlying the anesthesia induced reduction in direction selectivity

GABAergic inhibition has been theorized to play a major role in the emergence of direction selectivity in cortical and retinal networks [104–107]. Blocking inhibitory inputs to pyramidal neurons in the primary visual cortex reduces direction selectivity by allowing a stronger response to the null direction [108,109], while it does not affect orientation selectivity [110]. In contrast, stimulating activity of Parvalbumin positive interneurons in the local V1 network leads to a sharpening of direction selectivity and tuning curve bandwidth [111]. The effects we observed, reduced direction selectivity and an increased response to the null direction, are consistent with an impairment of cortical inhibitory function in anesthetized animals [42]. This may seem unexpected because isoflurane, as other anesthetics, is known to potentiate inhibitory currents [12,112,113] and may be expected to lead to an increase rather than a decrease in direction selectivity. There are, however, many subclasses of GABAergic interneurons in the neocortex [114,115] and isoflurane-induced potentiation of GABA_A_ receptor mediated inhibition may therefore have unexpected effects on tuning and inhibition of cortical neurons. Parvalbumin-positive interneurons, for instance, which have been shown to sharpen direction tuning [111], receive significant inhibitory inputs on their proximal dendrites and soma [116] and may therefore be prevented from exerting this function under anesthesia.

Surround suppression in mouse primary visual cortex is mediated by somatostatin-expressing inhibitory neurons (SOMs) and is strongly reduced under Urethane anesthesia [43]. One could raise the hypothesis that changes in direction selectivity, as observed in our study, result from an iceberg effect of surround suppression on the preferred direction versus the null direction, which would be a manifestation of release of subtractive inhibition by SOMs under anesthesia [117]. A recent study of Lee et al. [118] confirms that SOMs provide subtractive inhibition in awake mice, but also shows that SOM activity is still able to suppress activity of pyramidal cells under isoflurane anesthesia, which was not the case under Urethane anesthesia. This anesthesia difference makes it difficult to directly ascribe the currently observed effect of less direction selectivity under anesthesia to reduced surround suppression by SOMs. Nevertheless, Haider et al. [42] reported a reduction in surround suppression for V1 pyramidal cells in isoflurane-anesthetized mice, compared to awake animals. Identity of the interneurons mediating this inhibition remained, however, unclear. Therefore, it is difficult to predict if the effect of such increased surround suppression on orientation tuning in the receptive field center would be subtractive and thereby modifying tuning properties like direction selectivity (ice-berg effect), or would be divisive and thus leave tuning curve shapes intact (see [117]). A direct comparison of surround suppression and visually induced SOM activity in awake mice with that in isoflurane-anesthetized mice will potentially shed more light on this matter, while a better understanding of the circuitry underlying direction selectivity is needed to resolve this question fully.

In addition to affecting GABAergic synapses, isoflurane anesthesia inhibits glutamatergic transmission [119], which may also cause an overall shift in the cortical inhibition/excitation balance. This could result in a generalized loss of local inhibitory control on cortical information processing and as a consequence, tuning curves of neurons in primary visual cortex could become more similar to the less selectively tuned part of their thalamic input (i.e. to the lesser tuned overall excitatory drive instead of the strongly tuned F1/F0 component; [120]). Feedback connections from higher cortical areas may also contribute to sharper direction tuning in awake animals by driving specific groups of inhibitory interneurons [121], while being impaired under anesthesia [54].

In conclusion, isoflurane anesthesia reduces directional information coded by single neurons and populations of mouse primary visual cortex. While correlation patterns during visual stimulation were a negative predictor of mutual information on stimulus direction, the decrease in direction coding could not be explained by anesthesia-induced changes in correlation patterns in general, but rather resulted from an increase in responsiveness to the null direction.

## Supporting Information

S1 Fig Variance in tuning curve parameter estimation for raw and fitted tuning curves at different signal to noise ratios. (A) Circular variance in estimation of preferred direction based on simulated raw tuning curves (Purple) or two-peaked circular Gaussian fits of those raw curves (Green) as a function of signal to noise ratio in simulated single trial responses. (B) Idem, but for preferred orientation. (C, D and E) Idem, but for variance in orientation selectivity index (OSI), bandwidth (half-width at 1/√2 height of the tuning curve) and direction selectivity index (DSI). Error bars represent 95% confidence intervals.
(TIF)

S2 Fig Direction selectivity of cells preferring different directions of movement. Mean (± SEM) direction selectivity index for cells grouped by their preferred direction, in 45° wide bins centered on 0°, 45°, till 315°. Red and blue indicate data from the awake and anesthetized recordings, respectively.
(TIF)

S3 Fig Visually evoked neuropil response. (A) Mean (± SEM) ΔF/F of the neuropil signal in response to visual stimulation, shown as a function of time (in seconds) for each movement direction separately (indicated by heading 45°, 90°, and so on). Time point zero indicates onset of the moving grating. Red and blue indicate data from the awake and anesthetized recordings, respectively. (B) Tuning curves of neuropil signals of individual recordings black (N = 10) and average (± SEM) across recordings in color. Left panel (red) shows awake data, right panel (blue) shows data from anesthetized recordings. (C) Same data as in B, but now realigned to the largest peak in the neuropil tuning curve per session. (D) Average ΔF/F signal across all neurons, stimuli and trials plotted against direction of movement (as in B). The average response across all neurons is relatively low because at any give moment, only a subset of the neurons is being stimulated by its preferred stimulus. (E) Same data as in D, but realigned to the largest peak of the neuropil tuning curve for each session. (F) Mean (± SEM) direction selectivity index for cells grouped by similarity of their preferred direction to the direction of the neuropil peak.
(TIF)

S4 Fig Awake-anesthesia differences in tuning curve parameters as a function of signal-to-noise ratio. Tuning curve parameters, quantified for groups of cells of decreasing size, which contained the neurons with the highest signal-to-noise ratio based on the ΔF/F responses to the preferred direction in the anesthetized condition. This resulted in progressively smaller groups having higher average signal-to-noise ratios. Group size was stepwise reduced from 110 neurons to 30 neurons; plotted on the x-axis. All data are presented as mean (± SEM) for (A) Direction selectivity index. (B) ΔF/F response to the preferred direction. (C) ΔF/F response to the null direction. (D) Orientation selectivity index. (E) Tuning curve bandwidth. (F-J) Same as A-E, but now with signal-to-noise ratio quantified using data from the awake condition. Red and blue curves indicate data from the awake and anesthetized recordings, respectively. The data in A-E and F-J can be compared to Fig. 3C-G respectively.
(TIF)
