# Supplementary figures and images for: Effects of Isoflurane Anesthesia on Ensemble Patterns of Ca2+ Activity in Mouse V1: Reduced Direction Selectivity Independent of Increased Correlations in Cellular Activity
Source: PLoS One. 2015 Feb 23;10(2):e0118277. doi: 10.1371/journal.pone.0118277 (PMC4338011; doi:10.1371/journal.pone.0118277)

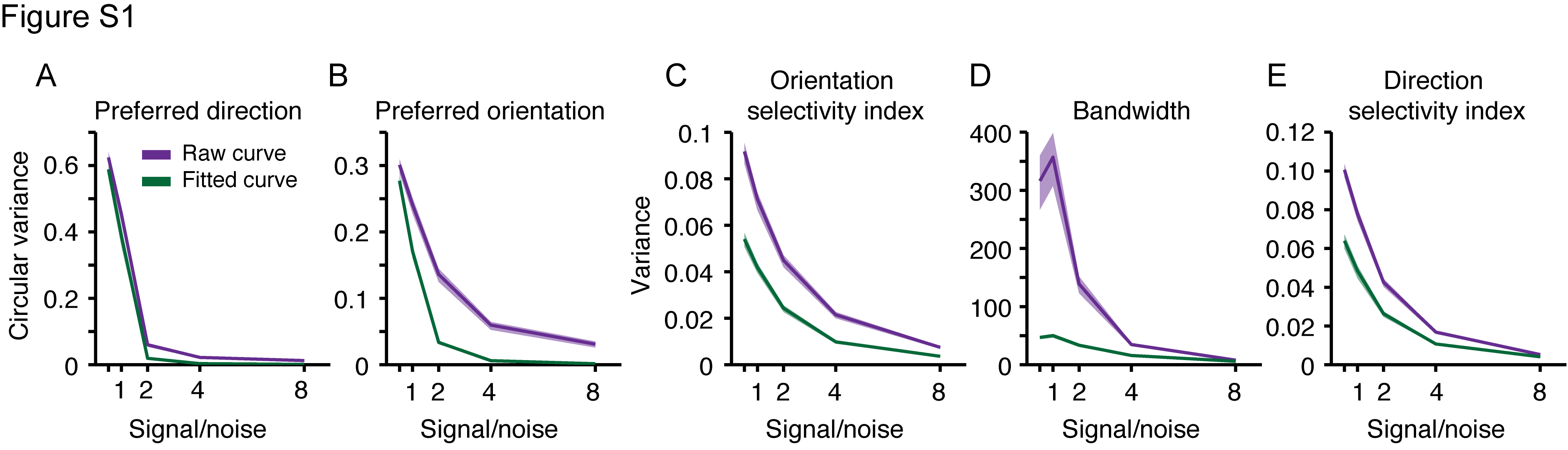

Supplement: S1 Fig — (A) Circular variance in estimation of preferred direction based on simulated raw tuning curves (Purple) or two-peaked circular Gaussian fits of those raw curves (Green) as a function of signal to noise ratio in simulated single trial responses. (B) Idem, but for preferred orientation. (C, D and E) Idem, but for variance in orientation selectivity index (OSI), bandwidth (half-width at 1/√2 height of the tuning curve) and direction selectivity index (DSI). Error bars represent 95% confidence intervals. (TIF) [file pone.0118277.s001.tif]

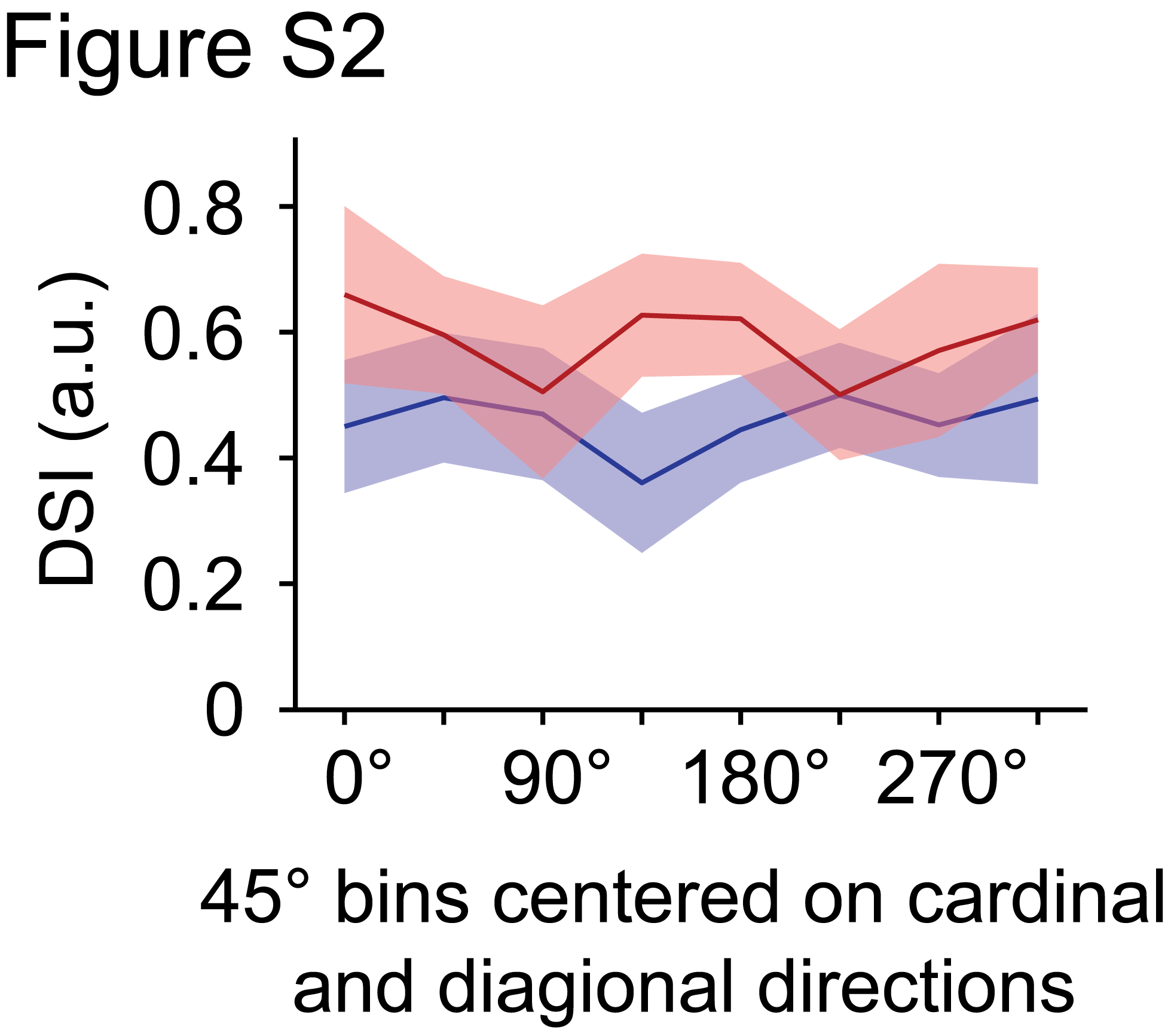

Supplement: S2 Fig — Mean (± SEM) direction selectivity index for cells grouped by their preferred direction, in 45° wide bins centered on 0°, 45°, till 315°. Red and blue indicate data from the awake and anesthetized recordings, respectively. (TIF) [file pone.0118277.s002.tif]

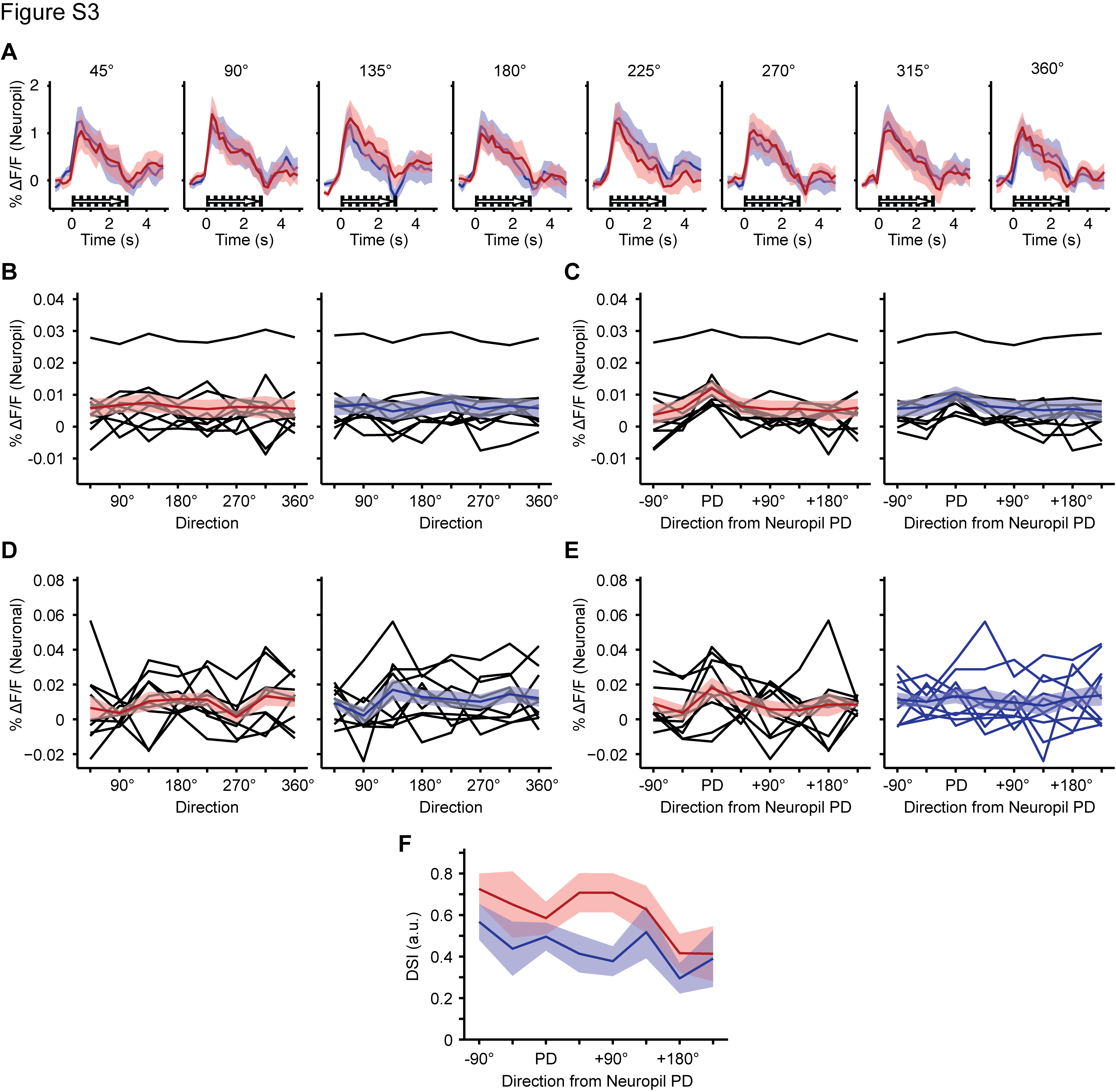

Supplement: S3 Fig — (A) Mean (± SEM) ΔF/F of the neuropil signal in response to visual stimulation, shown as a function of time (in seconds) for each movement direction separately (indicated by heading 45°, 90°, and so on). Time point zero indicates onset of the moving grating. Red and blue indicate data from the awake and anesthetized recordings, respectively. (B) Tuning curves of neuropil signals of individual recordings black (N = 10) and average (± SEM) across recordings in color. Left panel (red) shows awake data, right panel (blue) shows data from anesthetized recordings. (C) Same data as in B, but now realigned to the largest peak in the neuropil tuning curve per session. (D) Average ΔF/F signal across all neurons, stimuli and trials plotted against direction of movement (as in B). The average response across all neurons is relatively low because at any give moment, only a subset of the neurons is being stimulated by its preferred stimulus. (E) Same data as in D, but realigned to the largest peak of the neuropil tuning curve for each session. (F) Mean (± SEM) direction selectivity index for cells grouped by similarity of their preferred direction to the direction of the neuropil peak. (TIF) [file pone.0118277.s003.tif]

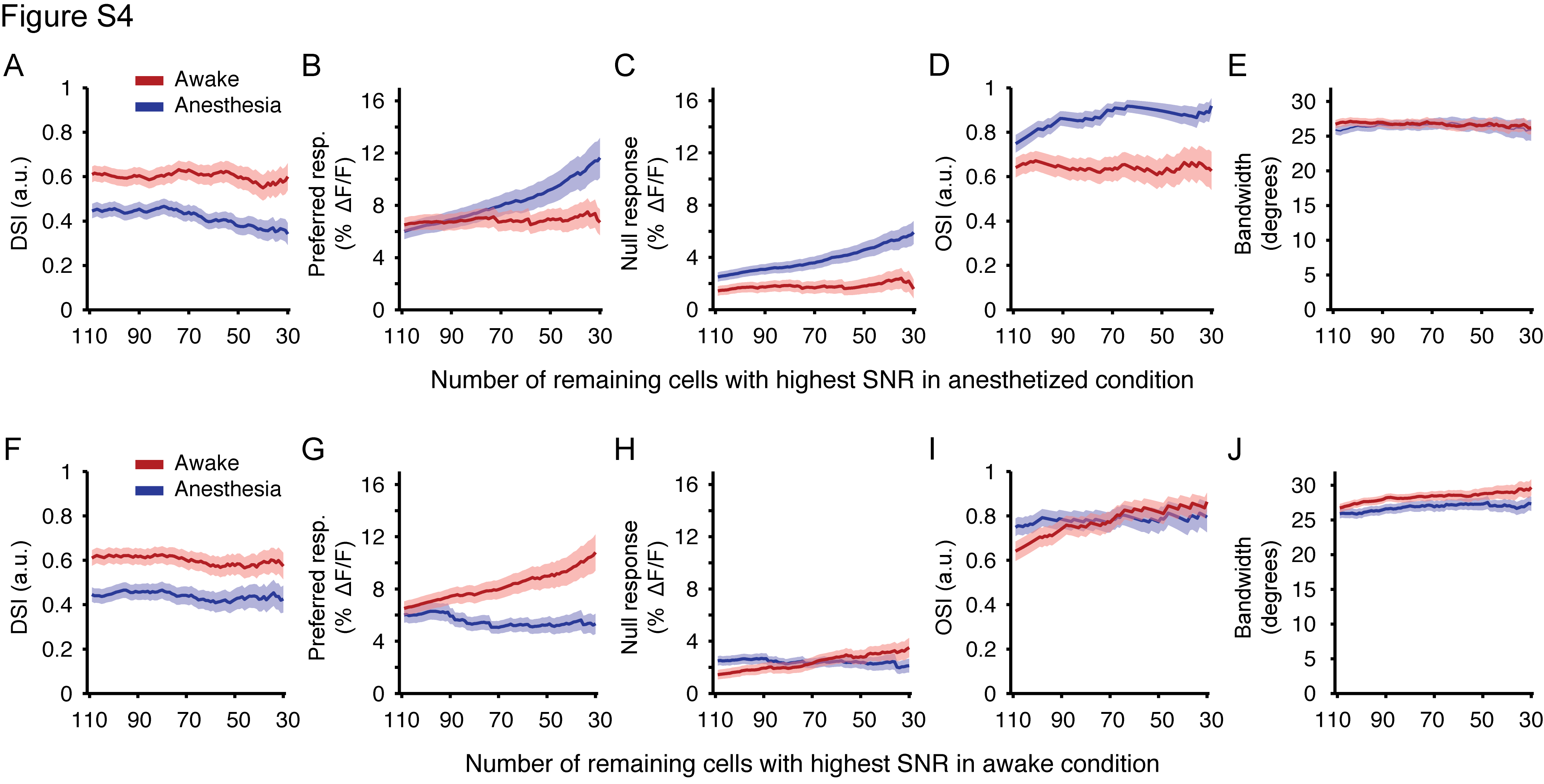

Supplement: S4 Fig — Tuning curve parameters, quantified for groups of cells of decreasing size, which contained the neurons with the highest signal-to-noise ratio based on the ΔF/F responses to the preferred direction in the anesthetized condition. This resulted in progressively smaller groups having higher average signal-to-noise ratios. Group size was stepwise reduced from 110 neurons to 30 neurons; plotted on the x-axis. All data are presented as mean (± SEM) for (A) Direction selectivity index. (B) ΔF/F response to the preferred direction. (C) ΔF/F response to the null direction. (D) Orientation selectivity index. (E) Tuning curve bandwidth. (F-J) Same as A-E, but now with signal-to-noise ratio quantified using data from the awake condition. Red and blue curves indicate data from the awake and anesthetized recordings, respectively. The data in A-E and F-J can be compared to Fig. 3C-G respectively. (TIF) [file pone.0118277.s004.tif]
